# Active Targeting Strategies for Improving the Bioavailability of Curcumin: A Systematic Review

**DOI:** 10.3390/foods14193331

**Published:** 2025-09-25

**Authors:** Yun-Shan Wei, Kun-Lun Liu, Kun Feng, Yong Wang

**Affiliations:** 1 College of Food Science and Engineering, Henan University of Technology, Zhengzhou 450001, China; 2 College of Food and Bioengineering, Henan Key Laboratory of Cold Chain Food Quality and Safety Control, Zhengzhou University of Light Industry, Zhengzhou 450001, China

**Keywords:** nanocurcumin, receptor-mediated cell targeting, colon-specific targeting, controlled release, bioavailability

## Abstract

Curcumin (CUR) is a bioactive compound with well-documented therapeutic potential in diverse pathological conditions, encompassing intestinal disorders—most notably colonic cancer—as well as extra-intestinal malignancies such as hepatic, breast, and renal tumors. However, the therapeutic efficacy of CUR is severely constrained by its poor aqueous solubility, chemical instability, and consequent low systemic bioavailability. Nano-scaled carriers (nanocurcumin) enhance CUR solubility and membrane permeability through their reduced dimensions and/or specific interactions with membrane constituents. Nevertheless, conventional nanocurcumin formulations, such as unmodified liposomes, nanocapsules, nanogels, and nanofibers, continue to accumulate substantially in non-target tissues because of their lack of disease-specific tropism. This review focuses on the most recent advances in active targeting strategies for nanocurcumin, specifically receptor-mediated cellular targeting for extra-intestinal pathologies and colon-specific ligand-directed delivery for intestinal disorders. Current methodologies for validating the efficacy of engineered nanocurcumin formulations are critically reviewed, and the prevailing limitations alongside prospective future applications of nanocurcumin are delineated and discussed.

## 1. Introduction

**Figure 1:**
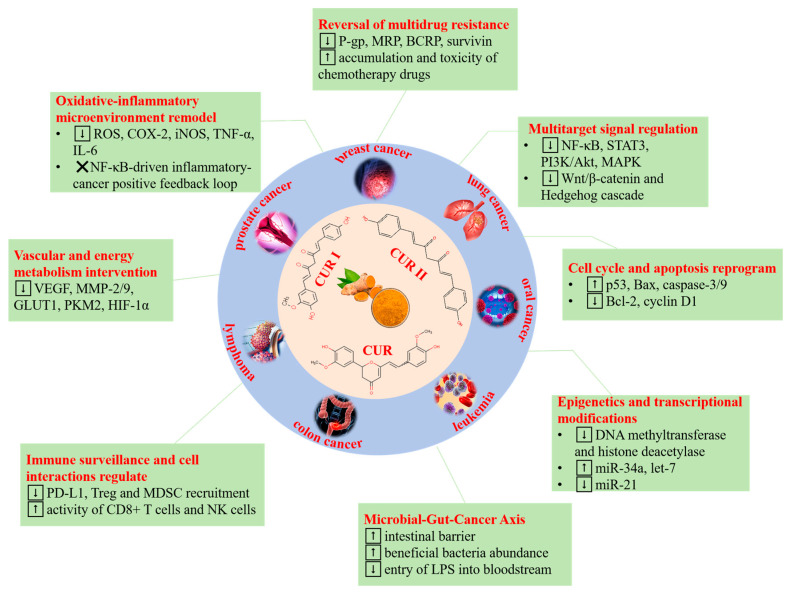
Schematic diagram of the multi-dimensional anticancer mechanism of curcumin (CUR: curcumin; CUR I: demethoxycurcumin; CUR II: bisdemethoxycurcumin; ↑, ↓ and ✕ mean upregulation, downregulation, and pathway blockade, respectively.).

Cancer, the leading global cause of morbidity and mortality, continues to exhibit a marked increase in incidence. Projections estimate that annual cancer-related deaths will reach 27 million by 2030 [1]. However, synthetic chemotherapeutics frequently exhibit limited efficacy, primarily attributable to the emergence of multidrug resistance, severe adverse effects, and progressive impairment of immune function. Consequently, there is growing interest in identifying novel natural products with anticancer activity, with particular emphasis on medicinal plants, botanical extracts, and plant-derived metabolites [2]. Curcuminoids were first isolated from turmeric in 1815 and comprise 77% diferuloylmethane (curcumin, CUR), 18% demethoxycurcumin (CUR II), and 5% bisdemethoxycurcumin (CUR III). CUR is the principal bioactive constituent of turmeric (Figure 1) and has been designated a third-generation cancer chemopreventive agent by the U.S. National Cancer Institute [3]. Consequently, this naturally occurring polyphenol represents a promising chemotherapeutic alternative for cancer management. Pre-clinical safety evaluations have demonstrated that CUR is well tolerated at high doses without observable adverse effects [4]. Furthermore, CUR has been shown to restore chemosensitivity, exerting anti-proliferative and pro-apoptotic effects in drug-resistant cancer cells while potentiating the cytotoxicity of conventional chemotherapeutic agents [5]. However, the clinical translation of CUR is severely impeded by its poor aqueous solubility (11 ng/mL), chemical instability under neutral to alkaline conditions, and extensive first-pass metabolism coupled with rapid systemic elimination, which collectively restrict its oral bioavailability [6,7].

**Figure 2:**
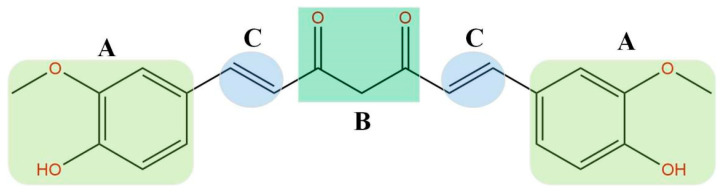
The curcumin chemical structure includes three main functional areas, i.e., two aromatic rings (part A, highlighted in yellow-green) linked to a β-dichetonic group (part B, highlighted in green) through a double bond (part C, highlighted in blue) [8].

The molecular structure of CUR comprises two phenolic rings connected via a heptadienedione linker containing a β-diketone moiety (Figure 2) [8]. The phenolic hydroxyl groups and the unsaturated β-diketone bridge are, however, regarded as critical liabilities that underlie CUR’s poor aqueous solubility, limited absorption, and rapid metabolic clearance [9]. To circumvent these drawbacks, numerous synthetic analogs, including diketone- and monoketone-modified structures as well as Knoevenagel condensation products, have been engineered [10]. Nevertheless, their mechanisms of action are inherently more intricate and remain a subject of conflicting reports.

**Figure 3:**
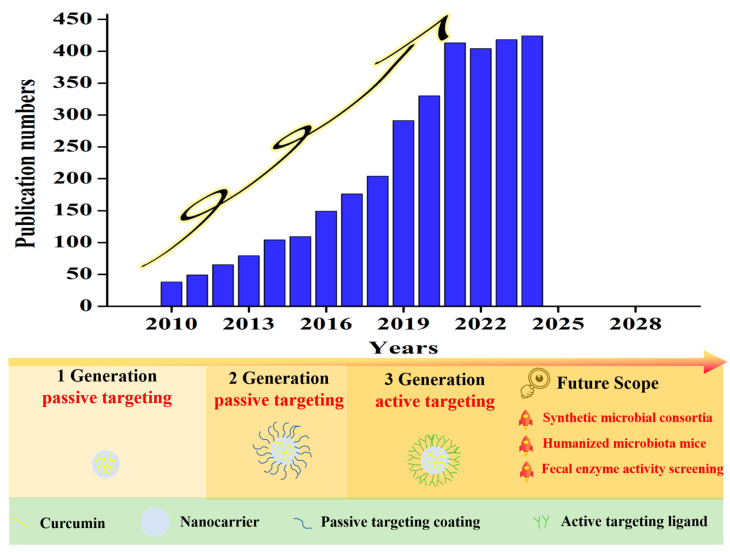
Annual publication counts retrieved from Web of Science using the keywords “curcumin” and “nanoparticle” (search date: 2010~2024), and the evolving research scope and future perspectives of curcumin-loaded nanoparticulate delivery systems [13]. First-generation passive targeting: The enhanced permeability and retention effect (EPR) of nano-scaled particles. Second-generation passive targeting: The nanocurcumin surface is engineered with a polymeric coating, typically polyethylene glycol, which establishes a densely hydrated shell that efficiently suppresses protein corona formation, evades reticuloendothelial clearance, and consequently amplifies tumor accumulation via the enhanced EPR effect. Third-generation passive targeting: Dominated by ligand–receptor interaction.

To circumvent the limitations inherent to CUR derivatives, the most extensively pursued strategy entails the encapsulation of CUR within nano-scaled delivery systems (nanocurcumin, Figure 3). Owing to their reduced dimensions and/or specific interactions with membrane constituents, these nanocarriers enhance transmembrane penetration while simultaneously mitigating CUR’s intrinsic hydrophobicity [11]. This approach has garnered substantial support from both academia and industry, as evidenced by the successful translation of several nanoformulations into clinically approved products marketed under brand names such as Myoce, Genexol-PM, Abraxane, Doxil (Caelyx), and DaunoXome [12]. Nevertheless, a significant proportion of these commercial formulations continues to exhibit substantial off-target accumulation attributable to their lack of disease-specific tropism.

Recent investigations have converged on two distinct active targeting paradigms tailored to specific pathological contexts. The first strategy addresses the non-specific accumulation of nanocurcumin in extra-intestinal malignancies by intravenously administering ligand-decorated nanocarriers that selectively engage receptors overexpressed on neoplastic cell surfaces [14]. For intestinal malignancies, particularly colorectal cancer, oral colon-targeted delivery systems are considered optimal [15]. Such systems protect CUR from degradation within the upper gastrointestinal tract and achieve high local concentrations at diseased colonic sites [16]. Design rationale is grounded in the distinct physiological and environmental differences that exist between the colon and the upper gastrointestinal tract [13].

This review therefore delineates the mechanistic rationale and most recent advances in nanocurcumin platforms that integrate either environment-responsive polymers or ligand-mediated active targeting, both of which enable site-specific tumor accumulation followed by controlled, sustained CUR release and thereby markedly enhance its bioavailability. Relevant analytical and pre-clinical methodologies for evaluating these systems are systematically summarized to elucidate their underlying delivery mechanisms. Finally, current limitations and prospective future directions for nanocurcumin are critically appraised and discussed.

## 2. Receptor-Mediated Targeting System

Direct interaction between CUR and cancer cells underpins its intrinsic antineoplastic activity [17]. Exploiting the overexpression of specific receptors or antigens on malignant cell membranes, surface-functionalized nanocarriers bearing targeting ligands have emerged as a promising strategy to augment cellular uptake and therapeutic efficacy of nanocurcumin [18]. This section first provides a comprehensive overview of the principal classes of ligands employed for nanocarrier decoration (Table 1) [19,20,21,22,23,24,25,26,27,28,29,30,31,32,33,34,35,36,37,38,39,40,41,42,43,44,45,46,47,48,49,50], including but not limited to nanocurcumin systems, followed by a detailed discussion of ligands specifically utilized for nanocurcumin surface modification.

### 2.1. Folic Acid

The dietary vitamin folic acid (FA) exhibits nanomolar affinity for the folate receptor, which is markedly overexpressed on a variety of cancer cells. Consequently, FA-decorated nanocarriers have been extensively investigated for their capacity to enhance CUR cellular uptake and systemic bioavailability [51,52]. FA is covalently conjugated to nanocurcumin surfaces via amide bond formation between FA’s γ-carboxyl group and reactive amine or hydroxyl moieties on the carrier. Reported coupling chemistries employ proteins (or polyamino acids), chitosan, anhydride- or imine-containing polymers, and graphene quantum dots as the surface-active scaffolds for this amidation reaction. For example, Kargar et al. [53] covalently tethered folic acid (FA) to a polycitric acid-grafted multi-walled carbon nanotube scaffold (MWCNT–PCA) via zero-length EDC/NHS amide chemistry. The FA ligand projects outward from the nanotube surface, enabling high-affinity recognition of overexpressed folate receptors on B16F10 melanoma cells. This folate-receptor-mediated endocytosis selectively increases CUR uptake while sparing normal, low-FA-receptor fibroblasts. Once internalized, the acidic endosomal milieu (pH ≈ 5.0) accelerates CUR release from the PCA layer, producing a 4.1-fold higher intracellular drug concentration and a 55% reduction in IC_50_ relative to ligand-free nanotubes, thereby potentiating the photothermal–chemotherapeutic efficacy of the MWCNT–PCA–FA/Cur platform under 808 nm laser irradiation. Inspired by recent advances, Dutta et al. [54] synthesized FA-decorated chitosan nanoparticles that entrap an indole–CUR analog through ionic gelation, yielding a 111 nm carrier with 98.7% encapsulation efficiency and pH-triggered release that potentiates cytotoxicity toward MDA-MB-231 cells. Concurrent encapsulation of Bcl-2 siRNA (Pol-CUR-siRNA-FA) suppressed drug efflux; the resulting nanosystem exhibited superior anti-HeLa efficacy through cell-cycle arrest, apoptosis, and autophagy induction. FA–chitosan conjugates, generated through the same amidation chemistry, have also been employed to encapsulate CUR-loaded magnetic bio-metal–organic frameworks (Fe_3_O_4_@Bio-MOF) and protein-based nanoparticles, markedly enhancing cellular uptake and cytotoxicity [55,56,57]. Molaei et al. [58] cleaved the anhydride rings of poly(maleic anhydride-alt-1-octadecene) (PMAO) and subsequently coupled FA through amide linkages to produce FA-modified nanocurcumin (MNPs). These MNPs displayed significantly higher toxicity toward MCF-7 and HeLa cells than free CUR. Zadeh et al. [59] synthesized Fe_3_O_4_–graphene quantum dot (GQD) hybrids that exploit the reaction between GQD carboxyl groups and FA amino groups to form amide bonds, thereby endowing the nanocurcumin with active cancer-cell-targeting capability. Collectively, these studies demonstrate that FA surface functionalization is a promising strategy for achieving precise CUR delivery to malignant cells.

### 2.2. Peptide

Owing to their low cytotoxicity, structural versatility, and intrinsic self-assembly capacity, peptides have emerged as highly attractive ligands for the construction of actively targeted nanocurcumin. A macrocyclic peptide Epi-1 that specifically binds EpCAM was grafted onto the surface of epirubicin/CUR co-loaded liposomes by Wang et al. [60]. This Epi-1 decoration accelerated receptor-mediated endocytosis in SKOV3 epithelial ovarian cancer cells, enabling a 2.5-fold higher intracellular drug accumulation and a 68% reduction in tumor volume compared with the non-targeted formulation. An integrin-targeting GRGDS peptide was covalently coupled to CUR-loaded magnetic PEGylated-PLGA nanoparticles; the resulting GRGDS-decorated nanocurcumin was found to exhibit a six-fold lower IC_50_ than its non-targeted counterpart in T98G glioblastoma cells after 72 h, confirming that active integrin binding accelerates intracellular CUR accumulation and cytotoxicity [61]. Likewise, Hou et al. [62] engineered M2pep-functionalized Mn-CUR metal–organic framework nanoparticles that specifically recognized M2-like fibrosis-promoting macrophages, and in a bleomycin-induced pulmonary fibrosis model, this M2pep-directed nanocurcumin depleted nearly 80% of the pro-fibrotic macrophages and reduced lung collagen content, illustrating the therapeutic advantage conferred by peptide-mediated cell-specific delivery. A tumor-responsive nanocurcumin was created by co-assembling a cystine-bridged peptide (CBP) with CUR [63]. Because GSH is overexpressed at tumor locations, the disulfide bond in the cysteine bridge is sensitive to the quantity of reduced/oxidized glutathione (GSH/GSSG). The produced nanocurcumin was thus able to quickly dissociate at tumor locations and inhibit tumor growth with few adverse effects on healthy tissues. A significant polypeptide ligand known as GE11 has the ability to specifically target colorectal cancer cells that have high epidermal growth factor receptor expression. Han et al. [64] created CUR-loaded nanocarriers more recently by taking advantage of the ability of hydrophobic proteins to self-assemble. The mentioned hydrophobic proteins are a broad family of proteins generated by filamentous fungi and include 8 conserved cysteine residues. Flexible linkers allow GE11 and nanocurcumin to be connected. In malignant cells, the anticancer compound CUR demonstrated greater accumulation and penetration, resulting in exceptional antitumor activity.

### 2.3. Antibody

The high antigen-binding affinity of antibodies toward tumor-overexpressed antigens underpins the rational design of antibody-functionalized nanocurcumin systems [65]. Annexin A2 (AnxA2) is markedly overexpressed in metastatic breast cancer. Exploiting this differential expression, AnxA2-targeted CUR-loaded PLGA nanoparticles (AnxA2-CPNP) were engineered. Comprehensive characterization demonstrated that, relative to non-targeted controls, AnxA2-CPNP exhibited markedly enhanced CUR delivery to metastatic breast cancer cells, resulting in pronounced inhibition of proliferation, migration, invasion, and neo-angiogenesis processes integral to tumor progression [66]. The epidermal growth factor receptor variant III (EGFRvIII), the most prevalent EGFR mutant, is expressed across multiple solid tumors, such as medulloblastoma, glioblastoma multiforme, breast, ovarian, prostate, and lung carcinomas, but is virtually absent from normal tissues. Jamali et al. [67] therefore engineered anti-EGFRvIII monoclonal-antibody-conjugated CUR-loaded PLGA nanoparticles (MAb-CUR-PLGA NPs). These targeted nanoparticles exhibited selective internalization into EGFRvIII-positive DKMG cells and elicited significantly higher photodynamic cytotoxicity than non-targeted controls (56% versus 24%). CD44, a transmembrane glycoprotein overexpressed in diverse malignancies, is a validated target for selective therapy. Demir et al. [68] incorporated CUR and carbon dots (CDs) as therapeutic and imaging payloads, respectively, into liposomes whose surfaces were further functionalized with anti-CD44 antibodies. The resulting actively targeted nanocarriers exhibited markedly enhanced antitumor efficacy and inherent fluorescence, underscoring their translational potential for theranostic applications. Rituximab is a chimeric mouse/human monoclonal antibody that selectively binds to the CD20 transmembrane antigen expressed in >95% of B-cell non-Hodgkin lymphomas (NHLs), the most prevalent adult hematological malignancy worldwide. Exploiting this selectivity, Varshosaz et al. [69] employed rituximab as a targeting ligand to construct CUR/imatinib co-loaded nanostructured lipid carriers (NLCs). The resulting targeted NLCs exhibited significantly higher cytotoxicity against CD20-positive Ramos B cells compared with free drugs or non-targeted NLCs, underscoring their potential for NHL therapy.

Beyond their high affinity for tumor-overexpressed antigens, antibodies possess intrinsic therapeutic potential that warrants consideration. Immune checkpoint blockade (ICB), which employs antibodies to neutralize programmed cell death-ligand 1 (PD-L1), has become a cornerstone of cancer therapy. Conjugating such therapeutic antibodies to immunoevasive nanodrugs can harness synergistic anticancer effects and yield superior therapeutic outcomes. For instance, Wang et al. [70] engineered an inflammation-responsive nanoplatform that co-delivers the NF-κB inhibitor CUR and a PD-L1 antibody, thereby reprogramming the tumor microenvironment (TME) and eliciting robust antitumor immunity in immunocompetent murine models. Likewise, anti-death receptor 5 (DR5) antibody represents a promising therapeutic for liver fibrosis. Antibody-mediated activation of death receptor 5 (DR5) triggers apoptosis of activated hepatic stellate cells (aHSCs), conferring antifibrotic efficacy. Nguyen et al. [71] synthesized an anti-DR5 antibody–CUR conjugate (DCC) via site-specific thiol–maleimide linkage between thiolated anti-DR5 and maleimide-functionalized CUR. DCC demonstrated pronounced hepatic tropism driven by antibody–DR5 interactions and, relative to the antibody alone, significantly reduced reactive oxygen species and inducible nitric oxide synthase in aHSCs, reflecting synergistic targeting and therapeutic effects. Consistently, in vivo pharmacokinetic analyses revealed preferential DCC accumulation in fibrotic livers.

### 2.4. Carbohydrate-Based Ligands

Hyaluronic acid (HA), a non-sulfated glycosaminoglycan composed of repeating disaccharide units, is abundant in the extracellular matrix. Its targeting utility stems from selective engagement with the transmembrane glycoprotein CD44—a receptor governing cell–cell interactions, adhesion, and migration—that is markedly up-regulated in malignancies [72]. Consequently, HA is exploited either as a direct drug conjugate or as a polymeric scaffold for nanocomplexes/nanoparticles to deliver hydrophobic chemotherapeutics or siRNA specifically to CD44-overexpressing cancer cells. Ghalehkhondabi et al. [73] constructed hyaluronic acid-decorated hollow mesoporous organosilica/poly(methacrylic acid) nanospheres that load CUR and respond synchronously to tumor acidity and intracellular glutathione. The carrier preferentially kills MCF-7 breast cancer cells (IC_50_ 38.5 µg/mL) while leaving normal-like cells largely unaffected, illustrating a cell-selective apoptotic effect driven by CD44-mediated uptake and dual-stimuli-triggered drug release. Wang et al. [74] constructed redox-sensitive HA-SS-PLGA micelles by tethering hyaluronic acid to PLGA through a cystine linker; the 198 nm vesicles stably encapsulated CUR (EE 70.8%) and released it rapidly (<83% in 48 h) when both tumor acidity (pH 5.4) and elevated glutathione (20 mM) were present, enabling CD44-directed delivery and amplified cytotoxicity toward MCF-7 breast cancer cells. Analogous HA-functionalized nanocurcumin systems have also been exploited to augment CUR’s topical anti-psoriatic efficacy and its therapeutic activity against osteosarcoma [75,76].

Hepatocellular carcinoma (HCC) is the predominant primary hepatic malignancy and characteristically overexpresses the asialoglycoprotein receptor (ASGPR) on its surface. Galactose (Gal) has therefore emerged as a potent targeting ligand, owing to its high-affinity interaction with ASGPR. Leveraging this specificity, Mokhtari et al. [77] engineered Gal-functionalized layered double hydroxide nanocarriers (Gal-Cur/LDH) for selective CUR delivery to HCC. Compared with free CUR and non-targeted LDH nanohybrids, Gal-Cur/LDH exhibited significantly enhanced cytotoxicity against HepG2 cells—an effect attributed to ASGPR-mediated uptake—while sparing ASGPR-low L929 normal cells.

Wang et al. [43] recently demonstrated that acetylation of konjac glucomannan (AceKGM) is pivotal for directing nanocarriers to macrophages. AceKGM selectively engages mannose receptors that are abundantly expressed on colonic macrophages, thereby enabling AceKGM-based nanocurcumin to target these cells and substantially enhance CUR uptake.

### 2.5. Glycoprotein/Glycosylamine

Lectins are carbohydrate-binding proteins distinguished by their exquisite glycan specificity. Plant lectins, in particular, are exploited to recognize aberrant glycosylation signatures on cancer cell membranes [78]. Covalent conjugation of tomato lectin to polystyrene nanoparticles increased their oral uptake 50-fold [79]. Galactosamine, another lectin-receptor ligand, was first employed by Sun et al. [80] to functionalize PEG-PLA/TPGS micelles for oral CUR delivery, and the resulting galactosamine-decorated micelles exhibited enhanced solubilization, receptor-mediated intestinal absorption, and markedly improved oral bioavailability.

Glucosamine (GlcN), an amino monosaccharide, targets tumors via high-affinity binding to the GlcN transporter—a glucose-transporting membrane protein overexpressed in malignancies such as breast cancer. Its abundant reactive functionalities allow facile conjugation to nanocarriers without compromising bioactivity [43]. Ghanbari et al. [81] synthesized CUR-loaded graphene quantum dots (GQDs) functionalized with GlcN. In MCF-7 cells, GlcN-mediated endocytosis conferred markedly higher fluorescence intensity and superior cytotoxicity compared with non-targeted CUR/GQDs, underscoring the enhanced CUR delivery potential of this multifunctional nanoassembly.

### 2.6. Combined Strategy

Dual-ligand nanoplatforms have been engineered to enhance CUR bioavailability and tumor specificity. Polysaccharide APS, isolated from Angelica sinensis, possesses well-documented hepatic tropism. Guo et al. [82] exploited this property by decorating nanocurcumin with both APS and glycyrrhetinic acid (GA), thereby generating a dual-targeted system that engages mannose and GA receptors on hepatoma cells. This construct exhibited markedly superior hepatic tumor accumulation compared with single-ligand analogs. Separately, although hyaluronic acid (HA) effectively targets CD44, dense HA clustering on nanocarrier surfaces may impede receptor function. To circumvent this limitation, Wang et al. [83] designed GA/HA dual-ligand nanocurcumin in which GA incorporation reduced HA surface density while preserving CD44-mediated targeting, thereby optimizing cellular response and therapeutic efficacy. Another emerging strategy involves engineering dual-targeted nanocurcumin systems that simultaneously engage CD44 receptors and exploit the pathological microenvironment. To diagnose and treat cerebral gliomas, Tian et al. designed redox-responsive micelles decorated with hyaluronic acid (HA). These carriers leverage the elevated glutathione (GSH) levels within tumor cells to cleave intramicellar disulfide bonds, thereby enabling rapid, site-specific CUR release [84]. Analogously, atherosclerotic plaques exhibit markedly higher reactive oxygen species (ROS) concentrations than normal tissues, and ROS-sensitive materials are thus advantageous for targeting such lesions. Accordingly, Dong et al. [85] developed a CD44-targeted nanomicelle that is also ROS-responsive, significantly enhancing the anti-atherosclerotic efficacy of CUR.

To enhance therapeutic efficacy and mitigate the adverse effects of conventional chemotherapy, nanocurcumin has been integrated with complementary clinical modalities. Malekmohammadi et al. [86] developed a multifunctional platform that couples FA-decorated nanocurcumin (CUR@PEI-FA-DSTNs) with sonodynamic therapy. Owing to folate receptor overexpression on HeLa cells, CUR@PEI-FA-DSTNs exhibited receptor-mediated endocytosis and markedly higher intracellular accumulation compared with A549 cells, resulting in pronounced cytotoxicity. Moreover, the combined chemo-sonodynamic regimen demonstrated significantly greater inhibition of tumor cell proliferation than either monotherapy alone.

## 3. Colonic Environment-Responsive Targeting

Colorectal cancer ranks as the second leading cause of cancer-related mortality worldwide [87]. Consequently, substantial efforts have been directed toward developing colon-targeted drug-delivery platforms that circumvent upper gastrointestinal degradation, hepatic first-pass metabolism, and systemic exposure, thereby enabling direct drug action at the disease site. Rational design of such systems exploits distinct physiochemical features of the gastrointestinal tract, namely pH gradients, enzymatic activities, and the colonic microbiota [88]. In our prior work, we systematically delineated these gastrointestinal characteristics and classified colon-targeted strategies into pH-dependent, enzyme/microbe-activated, and time-dependent modalities [89]. Current investigations of CUR colonic delivery predominantly focus on pH-responsive and enzyme-triggered nanocarriers.

### 3.1. pH-Responsive Delivery System

The pH along the human gastrointestinal tract progressively increases from the stomach to the distal ileum [90]. Exploiting this gradient, pH-responsive colon-targeted nanocurcumin systems have been engineered using enteric polymers that remain intact under acidic conditions and dissolve at the neutral-to-alkaline pH prevailing in the colon [91]. Among these, Eudragit^®^ (Evonik Industries, Eseen, Germany) is the polymer of choice. Khatik et al. [92] coated chitosan nanoparticles with Eudragit S100 (ES). In vitro and in vivo studies confirmed that the ES layer prevented premature CUR release in the upper gut while enabling maximal release in simulated colonic fluid, thereby enhancing systemic uptake and bioavailability. Lertpairod et al. [93] similarly employed ES coatings to suppress early leakage of CUR from nanostructured lipid carriers (NLCs). In a complementary approach, ES100 was grafted onto PLGA to create an amphiphilic, pH-sensitive copolymer that self-assembled into nanoparticles via emulsion–solvent diffusion [94]. Encapsulated CUR exhibited superior permeation across Caco-2 monolayers compared to free drug, and in vivo studies revealed reduced colonic myeloperoxidase activity and TNF-α release, confirming site-specific delivery. Mashaqbeh et al. [95] recently developed colon-targeted alginate/chitosan microbeads in which CUR was pre-encapsulated in *β*-cyclodextrin nanosponges and combined with 5-fluorouracil (5-FU). After five alternating layers of ethylcellulose/Eudragit S100 coating, the 1.1 mm beads released ≈66% 5-FU and ≈73% CUR over 24 h at pH 7.4, followed zero-order (5-FU) or first-order (CUR) kinetics, and reduced HCT-116 viability to ≈23% within 72 h. X-ray tracking in rabbits confirmed arrival in the colon within 7–9 h post-gavage, underscoring the clinical translational potential of this dual-drug, pH/time-dependent platform.

Beyond polyacrylate copolymers, pH-responsive octenyl-succinylated curdlan-oligosaccharide (MCOS) has been exploited for CUR and quercetin colonic co-delivery micelles. MCOS micelles 153 nm in size released only ≈8% CUR in gastric fluid (pH 1.2) yet delivered ≈79% in simulated colonic medium (pH 7.4) within 42 h. In a human fecal fermentation model, MCOS micelles boosted propionate production and enriched Bifidobacterium, underlining their dual role as a prebiotic and colon-targeted polyphenol shuttle [96]. Similarly, Moideen et al. employed natural polymers—pectin and skimmed milk powder (SMP)—to dual-coat CUR-loaded solid lipid nanoparticles (SLNs) [97]. The resulting dual-layer nanocurcumin exhibited superior colonic stability, a sustained release of 92.1% over 72 h in simulated gastrointestinal media, and enhanced drug loading. In vitro studies demonstrated a pronounced increase in CUR cytotoxicity against human colorectal adenocarcinoma (Caco-2) cells.

Conventional pH-responsive colonic systems are designed around the physiological pH of the healthy intestine and therefore dissociate only under the mildly alkaline conditions typical of the distal colon. However, the microenvironment of colonic tumors is acidic, rendering such platforms incapable of reliable, site-specific CUR release [85]. To address this limitation, Zhang et al. engineered programmed core–shell nanoparticles (CNs@EPO@L100) composed of an acid-responsive, CUR-loaded Eudragit^®^ EPO core enveloped by an Eudragit^®^ L shell (critical dissolution pH 6.0) [98]. These nanoparticles exhibited precisely programmed pH-triggered release, enhanced accumulation within inflamed colonic tissue, and superior anti-inflammatory activity in vitro. Oral administration of CNs@EPO@L100 markedly attenuated disease severity in a murine model of colon cancer.

### 3.2. Microbial/Enzyme-Sensitive Delivery System

The colonic lumen is enriched with hydrolytic and reductive enzymes, predominantly of gut microbiota origin, that can trigger site-specific release of bioactives [99]. In microflora-activated systems, these enzymes cleave carrier–drug linkages to liberate the therapeutic payload. Huang et al. [100] exemplified this strategy by synthesizing an α-amylase-responsive microcapsule, in which CUR was grafted onto hydroxyethyl starch through an ester linkage. In vitro release assays revealed <10% cargo leakage across simulated gastric and small-intestinal fluids. In DSS-provoked mice, these microcapsules preferentially accumulated in inflamed colons after oral delivery, markedly restored epithelial integrity and reduced splenomegaly, thereby validating their utility as an enzyme-activated combination platform for targeted ulcerative colitis therapy.

Non-digestible polysaccharides and their derivatives serve as both structural scaffolds and colonic gatekeepers for CUR release, yet their hydrophilic matrices necessitate strategies to accommodate CUR’s poor aqueous solubility. Li et al. [101] overcame this limitation by first forming CUR–cyclodextrin inclusion complexes, which were subsequently encapsulated within a polysaccharide shell composed of low-molecular-weight chitosan and unsaturated sodium alginate. Zhang et al. [102] employed coaxial electrospray to fabricate zein–shellac core–shell microparticles in which CUR was embedded within the zein core and simultaneously enveloped by a shellac coat. Zeybek et al. [103] precipitated negatively charged xylan onto anionic mesoporous silica nanoparticles via in situ chitosan polymerization, yielding a polysaccharide-gated nanocarrier. Wen et al. [104] utilized highly adsorptive porous starch gels to sequester CUR in the core, while a multilayered shell comprising chitosan, alginate, guar gum, and low-methoxyl pectin ensured site-specific release in the colon. Departing from the aforementioned strategies, Meng et al. fabricated a one-step, CUR-loaded konjac glucomannan octenyl succinate (KGOS) nanoemulsion that exploits the amphiphilicity and colon-specific degradability of KGOS [105]. Wang et al. further advanced emulsion-based colonic delivery by enveloping a whey-protein-isolate-stabilized CUR nanoemulsion with carboxymethyl konjac glucomannan (CMKGM), either alone or in combination with chitosan [106]. In a separate green-engineering approach, the same group employed supercritical-fluid-assisted atomization to generate CUR microcapsules from oil-in-water emulsions [107]. Colon-specific release was rigorously validated through in vitro dissolution under simulated gastrointestinal conditions and in vivo tracking or disease models. Specifically, Wen et al. observed minimal CUR release in upper-GIT media followed by substantial release in colonic fluids, accompanied by marked attenuation of dextran-sulfate-sodium-induced ulcerative colitis in mice and favorable modulation of inflammatory protein expression [104]. Zhang et al., using in vivo fluorescence imaging, confirmed the colonic accumulation and targeting efficiency of shellac-coated CUR/zein microparticles [102], and chronic oral administration of these microparticles for one week significantly mitigated acute experimental colitis compared with free CUR or CUR/zein particles alone. Additionally, probiotic-derived polysaccharides partially restored intestinal dysbiosis associated with colorectal cancer and reduced pathobiont burden [104].

Recent studies have integrated ligand-directed functionalization with microbiota-triggered release to enhance the cellular precision of colon-targeted nanocurcumin. Kolta et al. [108] engineered a polymeric hybrid whose surface was covalently modified with HA; relative to unmodified nanoparticles, HA decoration markedly increased cellular association and uptake. Exploiting the thermally induced unfolding of globular proteins, Borah et al. [109] thermally coagulated albumin to generate nanogels that simultaneously entrapped CUR within their hydrophobic cores. These nanogels were subsequently encapsulated in a folate-decorated hyperbranched starch shell, yielding core–shell carriers that resisted physiological and gastrointestinal digestion yet selectively induced early apoptosis in folate-receptor-positive HT29 human colon adenocarcinoma cells. A more comprehensive summary of the research progress on CUR colon targeted delivery systems is presented in Table 2.

## 4. Evaluation Approaches for Active Targeting Nanocurcumin

### 4.1. Traditional Evaluation Methods

#### 4.1.1. In Vitro Release Tests

The release kinetics of CUR from its nanocarriers govern both the onset of therapeutic concentrations and the overall pharmacokinetic profile. Consequently, rigorous in vitro release profiling is considered an indispensable step in the pre-clinical evaluation of any nanocurcumin formulation. Conventional assays typically simulate the physiological pH landscape encountered across healthy and pathological tissues: approximately 7.4 in normal extracellular fluids, ~6.8 within the tumor interstitium, and 5.0–5.5 in the intracellular compartments of malignant cells, where lactate accumulation driven by heightened glycolysis prevails [122]. Exploiting these gradients, investigators have demonstrated accelerated CUR liberation from pH-responsive carriers under acidic conditions, a phenomenon probably ascribed to protonation/deprotonation of carrier-incorporated functional groups such as carboxylates [123].

Nevertheless, release data generated under these simplified buffer conditions may not accurately predict in vivo performance. An ideal in vitro platform should additionally recreate the tumor microenvironment or the intricate physiology of the gastrointestinal tract, encompassing dynamic pH shifts, shear stress, luminal pressure, resident microbiota, complex enzymatic cascades, and dietary constituents, all of which can influence carrier integrity and drug dissolution. Furthermore, these parameters are themselves modulated by external variables such as diet, physical stress, and disease status, compounding the challenge of developing a universally accepted, standardized protocol. Despite these limitations, a spectrum of advanced models that attempt to incorporate one or more of these physiological cues has recently been reported; the most representative of these approaches are summarized in the following sections.

##### In Vitro Dissolution Tests

Dissolution testing constitutes the most straightforward approach for evaluating release profiles of colon-targeted systems. Four standardized apparatuses—basket, paddle, reciprocating cylinder, and flow-through cell—are routinely employed [124]. Media are sequentially adjusted to mimic the physiological pH–transit profile of the gastrointestinal tract: pH 1.0 for 2 h (gastric phase), pH 6.8 for 4 h (jejunal phase), and pH 7.4 (colonic phase) [125]. Release kinetics are subsequently analyzed using established mathematical models, including zero-order, first-order, Higuchi, Ritger–Peppas, and Weibull equations, to elucidate the underlying mechanisms of drug liberation [67].

##### Modified In Vitro Dissolution Tests

Conventional dissolution media are limited to buffered solutions of varying pH, which inadequately recapitulate the enzymatic complexity of the human gastrointestinal tract. Consequently, updated in vitro release protocols incorporate digestive enzymes (i.e., pepsin, pancreatin, and trypsin) to better approximate physiological conditions [126,127]. Under these conditions, cumulative release is directly proportional to carrier degradation, and enzyme-supplemented media consistently yield higher release rates than enzyme-free controls. Inclusion of microbial enzymes such as β-glucosidase and pectinase further mimics the colonic microflora, rendering these enzyme-enriched media a robust platform for evaluating colon-targeted systems, particularly those activated by gut microbiota. More recently, several studies have incorporated fresh rat cecal contents or human fecal slurries directly into the release medium [96,128]. In one comparative report, microspheres released ≈90% of their CUR within 24 h in medium containing 1% rat cecal contents, compared with 84% in pectinase-supplemented medium and 80% in enzyme-free controls [129]. These data underscore the necessity of selecting physiologically relevant media to accurately assess the colonic release behavior of bioactives from targeted nanocarriers.

#### 4.1.2. Cell Tests

The therapeutic promise of active-targeted nanocurcumin is most often interrogated through an integrated two-tier in vitro strategy that couples cellular uptake with downstream cytotoxicity. Initially, qualitative evidence of ligand-mediated internalization is captured by exposing monolayers of selected cancer cell lines to fluorescently tagged nanocurcumin and its non-targeted counterpart. Confocal laser-scanning microscopy, flow cytometry or wide-field fluorescence imaging then charts the time-dependent accumulation of carriers in peri-nuclear regions [130,131]. These images are complemented by quantitative read-outs, either residual fluorescence in lysed cells measured on a microplate reader or HPLC-based quantification of extracted CUR, to calculate intracellular drug payload and to confirm receptor specificity [132,133]. Once the uptake signature is established, the biological sequelae are gauged with the MTT colorimetric assay, comparing dose–response curves between malignant and non-malignant cells to derive selectivity indices. Although this pairing of uptake and viability metrics offers a rapid first screen, it remains an oversimplification: monolayer cultures lack the three-dimensional architecture, extracellular matrix crosstalk, and heterogeneous cell populations that define native tumors, frequently overestimating potency. Consequently, data generated from these conventional assays should be interpreted as preliminary proof-of-concept rather than definitive evidence of therapeutic advantage, and must be corroborated in more physiologically nuanced models.

#### 4.1.3. In Vivo Tests

In vivo testing remains the decisive checkpoint for asserting both the targeting fidelity and the therapeutic utility of nanocurcumin, yet the value of the data is inseparable from the limitations of the animals in which it is generated. For colonic delivery, carriers are first tracked in real time through the rodent, canine, porcine, guinea-pig or rabbit gut, species selected because their gastric emptying times, intestinal lengths and microbial diversities partially echo human parameters. High-frequency X-ray radiography, γ-scintigraphy, and near-infrared fluorescence reveal whether formulations withstand gastric acid, arrive at the caecum within their design window, and disperse their payload synchronously with the rise in local β-glucosidase activity [134,135,136]. Ex vivo segmental dissection then provides the spatial resolution needed to corroborate imaging data and to calculate targeting efficiency. When the question shifts from “where does the particle go?” to “does it shrink the tumor?”, athymic or syngeneic tumor-bearing mice become the default model. Once an intravenous or intraperitoneal dose is administered, calipers record three orthogonal diameters (length L, width W, height H) so that volume can be estimated either simplistically as (L × W^2^)/2 or more accurately as π/6 × L × W × H; parallel T2-weighted MRI, micro-SPECT or IVIS fluorescence not only affirms the anatomical location of the lesion but also quantifies accumulation in tumor versus liver or spleen [56,137,138,139]. Notwithstanding their convenience, these murine xenografts grow subcutaneously rather than orthotopically, possess a compressed extracellular matrix, and are infiltrated by immune cells whose cytokine repertoire diverges from that of human patients, factors that can systematically overestimate nanoformulation efficacy. Consequently, modern validation pipelines are progressively incorporating orthotopic or genetically engineered mouse tumors, human-microbiota-associated rats, and even humanized mouse hybrids to narrow the translational gap that conventional caliper-based tumor monitoring cannot bridge.

### 4.2. Emerging Evaluation Methods

While traditional 2D cell lines and rodent models have facilitated the initial screening of active-targeted nanocurcumin, their translational relevance is increasingly questioned. Monolayer cultures lack the three-dimensional (3D) architecture, extracellular matrix (ECM), and cellular heterogeneity that define native tumors, often resulting in overestimated cytotoxicity and poor correlation with in vivo responses [140]. Similarly, conventional rodent models exhibit fundamental differences in gut physiology, such as faster transit time, distinct mucus composition, and altered microbiota, compared to humans, which can skew the evaluation of colon-targeted delivery systems [141].

To bridge this gap, tumor-derived organoids have emerged as a physiologically relevant in vitro platform. These 3D self-organizing structures retain patient-specific mutations, stromal components, and spatial organization, enabling more accurate assessment of ligand–receptor interactions and drug penetration. For instance, CUR-loaded folate-decorated nanoparticles showed significantly lower uptake in colorectal cancer organoids lacking FR-α expression, highlighting the predictive value of organoid models in stratifying responder populations [142].

In parallel, microfluidic gut-on-a-chip devices replicate the dynamic luminal environment, including peristalsis, shear stress, and oxygen gradients, allowing real-time monitoring of nanoparticle adhesion, mucus penetration, and epithelial transport. A recent study demonstrated that CUR nanocapsules exhibited enhanced colonic retention under physiologically relevant flow conditions, a phenomenon not observed in static Transwell assays [143].

For in vivo validation, humanized mouse models, either engrafted with human immune cells or reconstituted with human microbiota, are increasingly employed to recapitulate human-specific immune responses and microbial enzymatic activity. In a DSS-induced colitis model, human microbiota-associated (HMA) mice receiving CUR-loaded amylose-based micelles exhibited elevated butyrate production and reduced IL-6 levels, correlating with improved epithelial barrier function, a response absent in conventional SPF mice [144]. Additionally, orthotopic tumor models derived from patient-derived xenografts (PDX) or CRISPR-engineered organoids implanted in situ provide a more realistic tumor microenvironment for evaluating targeting efficiency and therapeutic outcome [145].

Collectively, these next-generation models offer mechanistic insights unattainable through conventional assays and should be integrated into future evaluation pipelines to enhance the translational fidelity of nanocurcumin formulations.

## 5. Core Challenges of CUR Active Targeting

Despite the impressive pre-clinical efficacy reported for ligand-decorated nanocurcumin, multiple structural, biological and translational bottlenecks continue to impede their progression beyond animal proof-of-concept. A critical re-evaluation of these hurdles is therefore essential to define realistic clinical development paths.

### 5.1. Antibody-Based Targeting: Cost, Stability and Immunogenicity

Monoclonal antibodies remain the gold standard for high-affinity recognition of tumor-associated antigens. However, their integration into nanocurcumin platforms introduces several non-trivial drawbacks. First, the high cost of GMP-grade antibodies and the multi-step bioconjugation chemistry markedly inflate formulation expenditure, limiting industrial scalability [146]. Second, antibody–nanoparticle conjugates frequently suffer from impaired long-term stability. For example, thiol-maleimide or EDC/NHS linkages can hydrolyze in aqueous media, leading to premature ligand shedding and loss of selectivity during storage [147]. Third, systemic administration of antibody-coated carriers risks off-target immune activation. Fc-domain interaction with complement or Fc-γ receptors can trigger cytokine release or anaphylactoid reactions, as observed in phase I trials of anti-HER2 liposomes [148]. Finally, the human anti-mouse antibody (HAMA) response remains a concern for murine-derived fragments, necessitating costly humanization steps. Collectively, these issues justify the exploration of lower-molecular-weight alternatives (peptides, aptamers, metabolites) until full human antibodies can be produced economically and linked via chemistries that survive the gastrointestinal tract.

### 5.2. Translational Gap Between Proof-of-Concept and Validated In Vivo Efficacy

A survey of the recent literature reveals that more than two-thirds of pH- or enzyme-responsive CUR carriers have been characterized solely in conventional 2D buffers or static Transwell systems. While these setups demonstrate “on–off” release behavior in response to acidic pH or isolated bacterial enzymes, they fail to reproduce the dynamic shear, mucus turnover and microbiota heterogeneity encountered in vivo. For example, CUR-loaded Schiff-base micelles released >80% of their payload within 2 h at pH 5.5, yet in vivo imaging in mice showed <15% colonic deposition because rapid gastric emptying neutralized the pH trigger before arrival at the caecum [149]. Conversely, only a handful of formulations, principally those employing robust polysaccharide (amylase/pectinase) or azoreductase-cleavable linkers, have demonstrated reproducible therapeutic outcomes in DSS colitis or orthotopic tumor models [100,102,104]. A stringent distinction must therefore be made between proof-of-concept systems validated exclusively in vitro and translation-ready platforms whose performance has been corroborated in at least two independent animal models that incorporate disease-relevant endpoints (e.g., cytokine modulation, tumor regression, endoscopic score).

### 5.3. Microbiota Complexity and Inter-Individual Variability

The colonic lumen houses > 1000 bacterial species whose collective enzymatic repertoire (β-glucuronidases, azoreductases, nitroreductases, glycosidases) can cleave pro-drugs or polymeric carriers, triggering CUR liberation [99]. However, this ecosystem is highly variable across ethnicity, diet, antibiotic history and disease state. Metagenomic analysis of healthy Europeans versus IBD patients revealed a distinct difference in *Bacteroides thetaiotaomicron*, a key producer of endo-amylase accounting for the divergent CUR release patterns observed for the same starch-based nanocapsule across different recipients [150]. Moreover, probiotic supplementation or intermittent antibiotic use can transiently suppress enzyme activity, leading to erratic drug exposure. Current manuscripts seldom quantify baseline microbiota or monitor post-dose shifts. Consequently, enzyme-sensitive release kinetics reported in SPF mice may not translate to human cohorts. Future studies should incorporate fecal enzyme activity screening, synthetic microbial consortia [151] or human-microbiota-associated (HMA) rodents to generate pharmacokinetic data that better anticipate clinical variability.

### 5.4. Regulatory and Manufacturing Considerations

Beyond biology, regulatory agencies require scalable, reproducible processes with defined critical quality attributes for each ligand–carrier pair. Unfortunately, many academic formulations rely on laboratory-grade reagents (e.g., low-polydispersity PLGA not commercially available > 100 g scale) or multi-step surface reactions that suffer from batch-to-batch ligand density heterogeneity. The lack of harmonized in vitro–in vivo correlation models for ligand-mediated nanoparticles further complicates dossier preparation. Until these industrial and regulatory gaps are addressed, active-targeted nanocurcumin is likely to remain in the “high-potential” but pre-clinical category rather than achieving Investigational New Drug approval.

In summary, while ligand-directed delivery has undeniably enhanced CUR’s cellular specificity and colonic accumulation, its clinical trajectory hinges on solving the intertwined challenges of economic viability, biological robustness, microbiota unpredictability and regulatory scalability. And the extended patents related to CUR-mediated active targeting are presented in Table 3. Addressing these bottlenecks demands interdisciplinary programs that integrate medicinal chemistry, microbial ecology and process engineering, rather than incremental optimization of ligand density alone.

## 6. Conclusions

CUR, a naturally occurring polyphenol, exhibits broad pharmacological activities. However, its clinical translation is hampered by extremely low aqueous solubility and rapid systemic degradation, which collectively restrict absorption and limit bioavailability. Substantial efforts have therefore focused on nanotechnological strategies to surmount these limitations. Nanoformulated CUR (nanocurcumin) has been meticulously engineered to enable tumor-specific delivery and controlled release. To date, active targeting—comprising cellular and site-directed approaches—has emerged as the principal paradigm for enhancing CUR bioavailability. Although these advances are encouraging, more sophisticated investigations are required to fully exploit nanocurcumin’s therapeutic potential.

Although a wide array of cell-targeted nanocurcumin formulations have been devised to enhance cellular uptake, tissue selectivity, and therapeutic potency, the majority remain at the proof-of-concept stage and have been evaluated solely in pre-clinical models. Consequently, their safety profile in humans remains undefined. Moreover, integrated strategies that couple precise targeting with tumor microenvironment responsiveness require further exploration to achieve safe and efficient CUR bioavailability. Finally, most investigations rely exclusively on in vitro assays that inadequately predict in vivo performance. Therefore, a comprehensive evaluation framework incorporating in vitro, in vivo, and ultimately clinical assessments is imperative to rigorously determine the translational potential of nanocurcumin. 

Colon-targeted nanocurcumin systems traditionally rely on pH sensitivity, microflora/enzyme activation, or mucoadhesion. However, single-mechanism or single-polymer platforms often exhibit erratic colonic localization and release. Multi-mechanistic or multi-unit formulations have therefore emerged as more reliable alternatives. A further challenge lies in polymer selection: although synthetic carriers have demonstrated favorable release profiles, future work should prioritize natural, biocompatible polymers. Additionally, a robust evaluation framework is required. This includes refined dissolution media that accurately recapitulate human gastrointestinal conditions and advanced imaging modalities capable of tracking carrier transit and release along the entire GI tract, thereby enabling mechanistic insights unattainable with release data alone.

## Figures and Tables

**Table 1:** Ligands for nanocarrier surface functionalization and recent advances in active targeting of cancer cells.

	Targeting Ligand	Overexpressed Receptor	Targeted Cells	Nanocarrier (NC)	Effect	References
Antibody	Trastuzumab	HER-2	Breast cancer cells (BT-474 cells)	CUR nanoparticles prepared by wet milling–solvent evaporation process	Improved cell diagnosis	[19]
	Anti-CD326	mAb-CD326	Breast cancer tumors	Glutathione-stimulated responsive nanocarrier	Improved cell targeting capacity	[20,21,22,23]
	Single-chain fragment variable (scFv) antibody	VP28	White spot syndrome virus (WSVV)	Carbon nanotubes		
	Anti-HER2-FITC	Anti-HER2-FITC antibody	Several tumor cells	Lipid liquid nanoemulsion		
	PSMA monoclonal antibody	PSMA	Prostate cancer tumor cells	Peptide-based conjugated nanoparticles		
	αDEC205 antibody	αDEC205	Dendritic cells	Bacteria-derived outer membrane vesicles	Remodeled dendritic cell uptake pattern	[24]
Growth factor	EGF	VEGF receptor	Human A-431 tumor cells	Lipodisks	Improved cell targeting capacity	[25,26,27,28]
	Cetuximab	VEGF receptor	Mutant KRAS PANC-1 tumors	Lipodisks		
	K237/RGD/cRGD/LyP-1/Bombesin	Integrin/Annexin1/Integrin β6	Glioma/melanoma/ovarian cancer	Chitosan nanoparticles/polytyrosine nanoparticles		
	Plectin-1-targeting peptide	Plectin-1	Pancreatic tumor cells	Peptide nanoparticle platform		
Peptide/Protein	Cyclic RGD	Integrins	Various types of cancer cells	Liposome	Improved blood–retinal barrier permeability	[29]
	GE11 Peptides	Epidermal growth factor receptor (EGFR)	Liver tumor SMMC7721 cells	Polymersome/liposome	Increased cellular accumulation and antitumor activity	[30,31,32,33,34,35,36,37,38,39,40,41,42]
	Octreotide	Somatostatin receptor	MCF-7 cell lines	Silver nanoparticles		
	Cell-penetrating peptides (e.g., Penetratin, Polyarginine, Transportan, Pep-7, HIV-1TAT)	TFR	Numerous cancer cells	Liposome		
	Tumor-homing peptide	Neuropilin-1	Tumor blood vessel	Lipid–polymer hybrid nanoparticles		
	Transferrin	Transferrin receptor	Tumor in intracranial orthotopic models/gliomas	DSPE-PEG2k nanoparticles/PEGylated liposome		
Aptamer	Lectin	Cell surface glycans	Glioblastoma phenotype astrocyte cells	Lectin-coated fluorescent nanodiamonds		
	Anti-EGFR aptamer	EGFR	Various tumor cells	DNA nanotube framework		
	AS1411	Nucleolin receptor	Breast cancer cell	Silver nanotriangles		
	Mucin 1 (MUC1) aptamer	MUC1	Lung cancer cells	PLA-PEG nanocarriers		
Vitamin	Folic acid	Folic acid receptor	Various tumor cells	Liposome		
	Biotin	biotin receptor	Hepatocellular carcinoma	Brush copolymer nanocarrier		
Sugar	Galactose	Asialoglycoprotein receptors	Various cancer cells	Micelles, polymeric nanoparticles, SLN, liposomes, etc.		
	Hyaluronic acid	Glycoprotein CD44		Cu-doped zeolite imidazole framework-8		
	Acetylated konjac glucomannan	Mannose receptors	Macrophages	AceKGM nanoparticles	Improved colonic macrophage targeting	[43]
Glycosylamine	Glucosamine	Glucose transporter 2	Breast cancer cells	Mesoporous silica-coated magnetic nanoparticle	Increased tumor targeting and MRI visualization	[44]
	N-acetyl-glycamide		Lung cancer cells	Micelles	Increased cellular accumulation and antitumor activity	[45,46,47,48,49,50]
Dual/multi-ligand	Glycyrrhetinic acid and peanut agglutinin	Glycyrrhetinic acid receptor, MUC1	Hepatoma carcinoma cells	Liposomes		
	Folic acid and lactobionic acid	asialoglycoprotein receptors and folate receptors		Galactosylated chitosan nanoparticles		
	Anti-carbonic anhydrase IX (anti-CA IX) antibody and CPP33	Carbonic anhydrase IX	Lung cancer	Liposome		
	Anti-CD133 and folic acid	CD133 and folate receptor	Colon cancer cells	Hybrid biomimetic nanomedicine		
	Hyaluronic acid, SYL3C and CL4	CD44/epithelial cell adhesion molecule/EGFR	Stem tumor cells/epithelial cancer cells	Multi-targeting nanosystem		

**Table 2:** Additional representative research advances in colonic environment-responsive targeting strategy.

Category	Targeting Stimuli	Formulation Strategy	Key Findings	References
pH-responsive	Gradual pH increase from stomach to colon	Core–shell microparticles with shellac (pH-sensitive polymer) coating	Shellac coating delayed CUR release before colon Burst release occurred at colonic pH Enhanced colonic accumulation and anti-inflammatory efficacy in DSS-induced colitis mice	[102]
		Carboxymethyl curdlan–quercetin conjugate-stabilized Pickering emulsion	Stable in acidic gastric fluid Released CUR in simulated colonic fluid	[110]
	Tumor microenvironment (acidic pH)	CUR-loaded aerogel functionalized with pH-responsive cell-penetrating peptide (PLP) and coated with trehalose	Trehalose coating prevented drug release in acidic pH PLP enhanced cellular uptake at tumor site Showed superior cytotoxicity against HT29	[111]
Microbiota-active	Gut microbiota metabolism	Curcumin and resveratrol-co-loaded Mesona chinensis polysaccharides/zein nanoparticles	Enhanced gastrointestinal stability Promoted short-chain fatty acid production Modulated gut microbiota to alleviate UC	[112]
	Xylanase-producing *Bacteroides* spp.	Xylan–chitosan-coated P-123 micelles	≤85% CUR retained in upper GIT; 27–33% triggered release by *B. ovatus* in colon	[113]
Enzyme-responsive	Anaerobic azo-reductase	Mixed micelles	76% CUR released in rat colon fluid Strong mucoadhesion and anti-UC efficacy	[114]
	Pectinase in colon	Bilayer W1/O/W2 emulsion with pectin outer shell	90% CUR in colon	[115]
		Pectin–chitosan hydrogel encapsulating folate-modified CUR liposomes (FA-NLC@MPs)	FA-NLC@MPs protected CUR in upper GI tract Pectin degraded in colon, releasing FA-NLC for CD44-targeted uptake and enhanced colitis treatment	[116]
	α-amylase overexpressed in UC mucosa	Hydroxyethyl starch–CUR microspheres	80% cargo released in α-amylase-rich colon fluid 2.5-fold higher accumulation in inflamed colon	[117]
	β-glucanase in colon	Mucoadhesive tablets using jackfruit–okra mucilage blend	Tablets remained intact in stomach and small intestine Swelling and degradation occurred in colon due to microbial enzymes Enhanced CUR release in presence of cecal content	[118]
Redox-responsive	Inflamed colon environment (elevated ROS, α-amylase)	Microcapsules based on hydroxyethyl starch–CUR	Microcapsules released drugs in response to α-amylase in colon Preferentially accumulated in inflamed tissue via EPR effect Significantly reduced inflammation and restored colon length in DSS-induced colitis mice	[100]
pH/mucoadhesive dual-responsive	pH gradient and mucus layer adhesion	CUR-loaded microparticles using Eudragit^®^ FS and polycaprolactone	Colon-specific release Reduced oxidative stress and inflammation in acetic acid-induced UC rat model	[119]
Microbiota/enzyme dual-responsive	Colonic microbiota and pectinase enzymes	Low-methoxyl pectin (LMP) prepared through hydrostatic pressure-assisted enzymatic reaction (HHP) gelled with calcium	HHP-LMP beads resisted premature release in upper GI tract Degraded by colonic enzymes, enabling site-specific release	[120]
	β-glucanase and microbial fermentation in colon	Yeast cell wall β-glucan capsules + alginate/chitosan layer (“bionic yeast”)	β-glucan core resists gastric digestion Enzymatic degradation in colon, sustained 50% CUR release at 4 h and 14-fold higher local concentration vs. CUR	[121]
pH/enzyme dual-responsive	Colonic pH and pectinase	Guar gum-low methoxyl pectin/alginate–chitosan microgels loaded with porous starch–CUR	24 h colon retention F/B ratio restored TNF-α in DSS mice	[104]

**Table 3:** Patents on curcumin active delivery (cell-targeting patents are scarce; therefore, patents published or granted since 2010 are listed). Colon-targeting patents granted within the last five years are included.

Type	Patent Number	Legal State	Key Innovation Points	Evaluation	References
Activing targeting to cancer cells	US20100290982A1	Publication	CUR-NPs prepared by a novel S-O/W emulsion–diffusion–evaporation method. Dual-functional crosslinker conjugates CUR-NPs to targeting ligands for active tumor targeting.	In vitro uptake and cytotoxicity in breast cancer cell lines	[152]
	US20120183621A1	Publication	Nanocurcumin prepared via flash nanoprecipitation. Dual active CD44/CXCR4 targeting via DV3 surface modification of nanocurcumin.	In vitro cellular uptake, cytotoxicity, apoptosis, and metastasis inhibition assays In vivo orthotopic lung cancer mouse model	[153]
	US20130245357A1	Publication	Loading CUR onto magnetic nanoparticles enables targeted delivery to tumor tissues under the guidance of an external magnetic field.	In vitro cell uptake, cytotoxicity In vivo biodistribution, anti-tumor efficacy.	[154]
	US09023395B2	Grant	CUR-loaded PLGA NPs prepared via optimized double-emulsion solvent evaporation. Surface functionalized with bis-succinimidyl suberate and conjugated with Thy-1 antibodies for targeting.	In vitro uptake studies	[155]
	US20170202783A1	Publication	CUR was loaded in amphiphilic peptide nanoparticles self-assembled from C18GR7RGDS peptide. RGDS motif targets αvβ3 integrins on osteosarcoma and other cancers.	In vitro cell uptake, cytotoxicity, and selectivity	[156]
	US20180280517A1	Publication	Hydrophobic glycosaminoglycans form nanoparticles encapsulating CUR through self-assembly. The cancer cell-targeting molecule (e.g., EGFR antibody, CD-133 antibody, PD-L1 antibody) is bound to the nanoparticle through a crosslinking agent.	In vitro cytotoxicity assay In vivo tumor growth inhibition	[157]
	CN110051855A	Grant	CUR, as a copolymer monomer, is used to construct prodrug structures to achieve extremely high drug loading capacity. ROS-responsive cleavage of the oxalate ester bond enables precise drug release upon H_2_O_2_ stimulation.	In vitro release under H_2_O_2_ and/or NIR light	[158]
	CN111686249A	Grant	GSH-responsive DSPAMAM dendrimer was integrated with gold nanorods (GNRs) featuring exceptional optical properties. GNRs further encapsulated with a mesoporous silica shell to sequester residual cetyltrimethylammonium bromide and to load CUR. RGD peptide was conjugated to the targeting ligand, enabling efficient cancer diagnosis and synergistic chemo-gene combination therapy.	In vitro cytotoxicity	[159]
	US20210236612A1	Publication	CUR was encapsulated in edible plant-derived exosome-like nanoparticles (EPELNs). Active targeting achieved by coating EPELNs with plasma membranes derived from tumor cells.	In vivo assay: assessing the inhibition of tumor growth and metastasis	[160]
	CN116731325A	Publication	An amphiphilic block copolymer (HA-b-PCDA) including poly(curcumin dicarboxylic anhydride) (hydrophobic segment) and hyaluronic acid (hydrophilic block) was prepared through a robust amide bond/. HA-b-PCDA exhibits dual CD44 targeting of tumor and cancer stem cells.	In vitro cellular uptake and cytotoxicity	[161]
	CN116925337B	Publication	A dopamine-derivative-based nanocarrier encapsulating CUR was constructed. Dopamine-derivative-based nanocurcumin extended CUR solubility and half-life. Dopamine-derivative-based nanocurcumin targeted FA, a receptor overexpressed in cancer cells.	In vitro drug release profiles at different pH values	[162]
	US20240226027A1	Publication	CUR-loaded injectable hydrogel nanoparticles fabricated via self-assembly of oleic acid-conjugated alginate. 100% drug loading achieved without any excipients. Trastuzumab was covalently linked to the gel surface via EDC/NHS chemistry for breast cancer targeting.	In vivo evaluation using SKBR-3 xenograft mouse model.	[163]
Colon targeted delivery	CN111000799B	Grant	Soft solid particles formed from ovalbumin–carboxymethyl konjac glucomannan complexes crosslinked with dihydromyricetin. CUR was loaded in a Pickering emulsion stabilized by the soft solid particles.	In vitro simulated gastrointestinal release assay In vivo oral administration in mice, assessing relative bioavailability of CUR	[164]
	CN111096950B	Grant	CUR-loaded double-layer emulsion was constructed using whey protein isolate (WPI) and carboxymethyl konjac glucomannan (CMKGM) as the inner/outer emulsifiers. The outer CMKGM layer provides enzyme-responsive targeting, while the inner WPI layer offers emulsifying stability and pH-sensitive protection.	In vitro simulated gastrointestinal release assay In vivo oral administration in mice, assessing relative bioavailability of CUR	[165]
	CN115364051B	Grant	A novel amphiphilic octenyl succinic anhydride-modified curdlan oligosaccharide (OSA-COS) was synthesized as a nanocurcumin. The system showed colon-targeted release due to dual triggers: pH change and microbial degradation in the colon.	In vitro pH-dependent release assay In vitro fecal fermentation, indicating microbiota-triggered release and prebiotic-like effects	[166]
	CN115364054B	Grant	A novel oil-in-water Pickering emulsion was developed using shellac nanoparticles and chitosan as stabilizers. CUR was encapsulated into shellac nanoparticles via antisolvent precipitation. The system enabled colon-targeted release and alleviated ulcerative colitis symptoms.	In vitro pH-dependent release assay In vivo oral administration in DSS-induced ulcerative colitis mice, assessing disease activity index	[167]

## Data Availability

No new data were created or analyzed in this study.

## References

[1] Fu Z.Y., Wang S., Zhou X., Ouyang L., Chen Z., Deng G. (2025). Harnessing the power of traditional Chinese medicine in cancer treatment: The role of nanocarriers. Int. J. Nanomed..

[2] Kah G., Chandran R., Abrahamse H. (2023). Curcumin a natural phenol and its therapeutic role in cancer and photodynamic therapy: A review. Pharmaceutics.

[3] Hewlings S.J., Kalman D.K. (2017). Curcumin: A review of its effects on human health. Foods.

[4] Hussain Y., Islam L., Khan H., Filosa R., Aschner M., Javed S. (2021). Curcumin-cisplatin chemotherapy: A novel strategy in promoting chemotherapy efficacy and reducing side effects. Phytother. Res..

[5] Akbari S., Kariznavi E., Jannati M., Elyasi S., Tayarani-Najaran Z. (2021). Curcumin as a preventive or therapeutic measure for chemotherapy and radiotherapy induced adverse reaction: A comprehensive review. Food Chem. Toxicol..

[6] Chen C.Y., Wang Z.X., Fu H.L., Yu G.Q., Luo X., Zhu K.W. (2024). Enhanced bioavailability of curcumin amorphous nanocomposite prepared by a green process using modified starch. Int. J. Biol. Macromol..

[7] Fan Y.T., Gan C., Zhang H.L., Yi J. (2024). Characteristics, physicochemical stability and in vitro release of curcumin-loaded glycated bovine serum albumin nanofibrils: Effects of molecular weight of saccharide. Food Hydrocol..

[8] Chen Z.F., Liu W.L., Zeng Z.J., Yan Z.H., Ma L.H., Liu Y., Cao X.S. (2025). Construction of active-passive dual-targeted drug-loaded micelle nanoparticles with modified dopamine molecules for efficient anti-tumor therapy. Int. J. Nanomed..

[9] Bayomi S.M., El-Kashef H.A., El-Ashmawy M.B., Nasr M.N.A., El-Sherbeny M.A., Badria F.A., Abou-Zeid L.A., Ghaly M.A., Abdel-Aziz N.I. (2013). Synthesis and biological evaluation of new curcumin derivatives as antioxidant and antitumor agents. Med. Chem. Res..

[10] Kuzminska J., Szyk P., Mlynarczyk D.T., Bakun P., Muszalska-Kolos I., Dettlaff K., Sobczak A., Goslinski T., Jelinska A. (2024). Curcumin Derivatives in Medicinal Chemistry: Potential Applications in Cancer Treatment. Molecules.

[11] Wilar G., Suhandi C., Fukunaga K., Shigeno M., Kawahata I., Abdulah R., Sasaki T. (2025). Effects of nanocurcumin supplementation on metabolic syndrome: A systematic review and meta-analysis of randomized controlled trials. Pharmacol. Res..

[12] Ahmad E., Ali A., Fatima M.T., Nimisha, Apurva, Kumar A., Sumi M.P., Sattar R.S.A., Mahajan B., Saluja S.S. (2021). Ligand decorated biodegradable nanomedicine in the treatment cancer. Pharmacol. Res..

[13] Wang Y.D., Li Z.Y., Bao Y.W., Cui H.J., Li J.X., Song B.G., Wang M.Z., Li H.K., Cui X.Y., Chen Y. (2025). Colon-targeted delivery of polyphenols: Construction principles, targeting mechanisms and evaluation methods. Crit. Rev. Food Sci. Nutr..

[14] Hegde M., Kumar A., Girisa S., Aswani B.S., Vishwa R., Sethi G., Kunnumakkara A.B. (2023). Nanoformulations of curcumin: An alliance for effective cancer therapeutics. Food Biosci..

[15] Wang L.C., Yu M., Yang H. (2021). Recent progress in the diagnosis and precise nanocarrier-mediated therapy of inflammatory bowel disease. J. Inflamm. Res..

[16] Kamath A.J., Donadkar A.D., Nair B., Kumar A.R., Sabitha M., Sethi G., Chauhan A.S., Nath L.R. (2025). Smart polymer-based delivery systems for curcumin in colon cancer therapy: A review. Phytother. Res..

[17] Azehaf H., Benzine Y., Tagzirt M., Skiba M., Karrout Y. (2023). Microbiota-sensitive drug delivery systems based on natural polysaccharides for colon targeting. Drug Discov. Today.

[18] Wang D.D., Zhang X.N. (2021). Advances in receptor modulation strategies for flexible, efficient, and enhanced antitumor efficacy. J. Control. Release.

[19] Yaman Y.T., Vural O.A., Bolat G., Abaci S. (2023). Fabrication of trastuzumab conjugated curcumin nanoparticles based impedimetric cytosensor for the cancer cell detection. Microchem. J..

[20] Li H.J., Zhang M.Z., He J.L., Liu J., Sun X.W., Ni P.H. (2023). A CD326 monoclonal antibody modified core cross-linked curcumin-polyphosphoester prodrug for targeted delivery and cancer treatment. J. Mater. Chem. B.

[21] Huang A.G., Chen C., Liu T.Q., Wang G.X. (2022). scFv antibody-mediated targeted drug delivery system improves the antiviral activity of geniposidic acid against WSSV. Aquaculture.

[22] Navarro-Marchal S.A., Martín-Contreras M., Castro-Santiago D., del Castillo-Santaella T., Graván P., Jódar-Reyes A.B., Marchal J.A., Peula-García J.M. (2023). Effect of the protein corona formation on antibody functionalized liquid lipid nanocarriers. Int. J. Mol. Sci..

[23] Liu Y., Hao L., Dong Y., Dong B.Z., Wang X.L., Liu X., Hu Z.X., Fang G.C., Wang G.Y., Qin J.X. (2024). Co-delivery of siape1 and melatonin by 125I-loaded PSMA-targeted nanoparticles for the treatment of prostate cancer. Recent Pat. Anti-Cancer.

[24] Liang J., Cheng K.M., Yao X., Chen Y.W., Ma N.N., Feng Q.Q., Zhu F., Ma X.T., Zhang T.J., Yue Y.L. (2022). Personalized cancer vaccines from bacteria-derived outer membrane vesicles with antibody-mediated persistent uptake by dendritic cells. Fundam. Res..

[25] Ahlgren S., Fondell A., Gedda L., Edwards K. (2017). Egf-targeting lipodisks for specific delivery of poorly water-soluble anticancer agents to tumor cells. RSC Adv..

[26] McDaid W.J., Greene M.K., Johnston M.C., Pollheimer E., Smyth P., McLaughlin K., Van Schaeybroeck S., Straubinger R.M., Longley D.B., Scott C.J. (2019). Repurposing of cetuximab in antibody-directed chemotherapy-loaded nanoparticles in EGFR therapy-resistant pancreatic tumors. Nanoscale.

[27] Ehrbar M., Rossi F., Cellesi F. (2020). Editorial: Nanosized drug delivery systems: Colloids and gels for site specific targeting. Front. Bioeng. Biotechnol..

[28] Wang Y.Z., Du C., Zhao Y., Nie G.J., Yang Y.M. (2020). Trap and kill strategy for nonBRCA mutant pancreatic cancer by codelivery of olaparib and JQ1 with plectin1 targeting peptide nanoparticles. Nano Today.

[29] Liu H., Zhang R., Zhang D., Zhang C., Zhang Z., Fu X., Luo Y., Chen S., Wu A., Zeng W. (2022). Cyclic RGD-decorated liposomal gossypol AT-101 targeting for enhanced antitumor effect. Int. J. Nanomed..

[30] Tang H.L., Pan Y.H., Zhang Y.F., Tang H.T. (2022). Challenges for the application of EGFR-targeting peptide GE11 in tumor diagnosis and treatment. J. Control. Release.

[31] Abdellatif A.A.H., Khan R.A., Alhowail A.H., Alqasoumi A., Sajid S.M., Mohammed A.M., Alsharidah M., Al Rugaie O., Mousa A.M. (2022). Octreotide-conjugated silver nanoparticles for active targeting of somatostatin receptors and their application in a nebulized rat model. Nanotechnol. Rev..

[32] An C.J., Wei S., Dao Y.K., Wang X.Y., Dong W.D., You X., Tian C., Zhang Z.L., Dong S.W. (2023). Discovery of endosomalytic cell-penetrating peptides based on bacterial membrane-targeting sequences. Bioorg. Chem..

[33] Gonzalez-Cruz A.O., Hernandez-Juarez J., Ramirez-Cabrera M.A., BalderasRenteria I., Arredondo-Espinoza E. (2022). Peptide-based drug-delivery systems: A new hope for improving cancer therapy. J. Drug Deliv. Sci. Technol..

[34] Li J.R., Zhang Z.X., Zhang B.L., Yan X.Y., Fan K.L. (2023). Transferrin receptor 1 targeted nanomedicine for brain tumor therapy. Biomater. Sci..

[35] Fard M.G., Khabir Z., Reineck P., Cordina N.M., Abe H., Ohshima T., Dalal S., Gibson B.C., Packer N.H., Parker L.M. (2022). Targeting cell surface glycans with lectin-coated fluorescent nanodiamonds. Nanoscale Adv..

[36] Baig M.M.F.A., Ma J.W., Gao X.L., Khan M.A., Ali A., Farid A., Zia A.W., Noreen S., Wu H.K. (2023). Exploring the robustness of DNA nanotubes framework for anticancer theranostics toward the 2D/3D clusters of hypopharyngeal respiratory tumor cells. Int. J. Biol. Macromol..

[37] Cao Y.Y., Yang H.Q., Li D.D., Li F., Ma J., Liu P.D. (2022). The effect of AS1411 surface density on the tumor targeting properties of PEGylated silver nanotriangles. Nanomedicine.

[38] Shahrad S., Rajabi M., Javadi H., Zarchi A.A.K., Darvishi M.H. (2022). Targeting lung cancer cells with MUC1 aptamer functionalized PLA-PEG nanocarriers. Sci. Rep..

[39] Mikled P., Chavasiri W., Khongkow M. (2024). Dual folate/biotin-decorated liposomes mediated delivery of methylnaphthazarin for anti-cancer activity. Sci. Rep..

[40] Varvarà P., Drago S.E., Esposito E., Campolo M., Mauro N., Calabrese G., Conoci S., Morganti D., Fazio B., Giammona G. (2024). Biotinylated polyaminoacid-based nanoparticles for the targeted delivery of lenvatinib towards hepatocarcinoma. Int. J. Pharm..

[41] Fatima M., Karwasra R., Almalki W.H., Sahebkar A., Kesharwani P. (2023). Galactose engineered nanocarriers: Hopes and hypes in cancer therapy. Eur. Polym. J..

[42] Zhang F., Cheng K., Zhang X.-S., Zhou S., Zou J.-H., Tian M.-Y., Hou X.-L., Hu Y.-G., Yuan J., Fan J.-X. (2024). Cascade-catalysed nanocarrier degradation for regulating metabolism homeostasis and enhancing drug penetration on breast cancer. J. Nanobiotechnol..

[43] Wang C., Guo Z.Z., Liang J.L., Li N., Song R.J., Luo L., Ai Y.L., Li X., Tang S.Q. (2022). An oral delivery vehicle based on konjac glucomannan acetate targeting the colon for inflammatory bowel disease therapy. Front. Bioeng. Biotech..

[44] Farjadian F., Faghih Z., Fakhimi M., Iranpour P., Mohammadi-Samani S., Doroudian M. (2023). Glucosamine-modified mesoporous silica-coated magnetic nanoparticles: A “Raisin-Cake”-like structure as an efficient theranostic platform for targeted methotrexate delivery. Pharmaceutics.

[45] Zhang S.Y., Li X., Liu Y., Li H., Zhang Z.Y. (2024). Physiologically driven nanodrug delivery system for targeted lung cancer treatment. Explor. Med..

[46] Li X., Diao W., Xue H., Wu F., Wang W., Jiang B., Bai J., Lian B., Feng W., Sun T. (2020). Improved efficacy of doxorubicin delivery by a novel dual-ligand-modified liposome in hepatocellular carcinoma. Cancer Lett..

[47] Xiang Y., Huang W., Huang C., Long J., Zhou Y., Liu Y., Tang S., He D.-X., Tan X.-W., Wei H. (2020). Facile fabrication of nanoparticles with dual-targeting ligands for precise hepatocellular carcinoma therapy in vitro and in vivo. Mol. Pharm..

[48] Lin C., Zhang X., Chen H., Bian Z., Zhang G., Riaz M.K., Tyagi D., Lin G., Zhang Y., Wang J. (2018). Dual-ligand modified liposomes provide effective local targeted delivery of lung-cancer drug by antibody and tumor lineagehoming cell-penetrating peptide. Drug Deliv..

[49] Zheng Y., Guo W., Hu L., Xiao Z., Yang X., Cao Z., Cao J. (2023). Long Circulating Cancer Cell-Targeted Bionic Nanocarriers Enable Synergistic Combinatorial Therapy in Colon Cancer. ACS Appl. Mater. Interfaces.

[50] Ren X.H., Han D., He X.Y., Guo T., Chen X.S., Pang X., Cheng S.W. (2023). Multitargeting nano-systems targeting heterogeneous cancer cells for therapeutics and biomarker detection. Adv. Healthc. Mater..

[51] Gao G.Y., Zhou W.H., Jiang X., Ma J. (2024). Bovine serum albumin and folic acid-modified aurum nanoparticles loaded with paclitaxel and curcumin enhance radiotherapy sensitization for esophageal cancer. Int. J. Radiat. Biol..

[52] Akbarzadeh I., Yaraki M.T., Ahmadi S., Chiani M., Nourouzian D. (2020). Folic acidfunctionalized niosomal nanoparticles for selective dual-drug delivery into breast cancer cells: An in-vitro investigation. Adv. Powder Technol..

[53] Kargar B., Fazeli M., Sobhani Z., Hosseinzadeh S., Solhjoo A., Akbarizadeh A.R. (2024). Exploration of the photothermal role of curcumin-loaded targeted carbon nanotubes as a potential therapy for melanoma cancer. Sci. Rep..

[54] Dutta D., Pajaniradje S., Nair A.S., Chandramohan S., Bhat S.A., Manikandan E., Rajagopalan R. (2024). An in-vitro study of active targeting & anti-cancer effect of folic acid conjugated chitosan encapsulated indole curcumin analogue nanoparticles. Int. J. Biol. Macromol..

[55] Kavya K., Vargheese S., Shukla S., Khan I., Dey D.K., Bajpai V.K., Thangavelu K., Vivek R., Kumar R.R., Han Y.-K. (2022). A cationic amino acid polymer nanocarrier synthesized in supercritical CO_2_ for co-delivery of drug and gene to cervical cancer cells. Colloids Surf. R Biointerfaces.

[56] Nejadshafiee V., Naeimi H., Goliaei B., Bigdeli B., Sadighi A., Dehghani S., Lotfabadi A., Hosseini M., Nezamtaheri M.S., Amanlou M. (2019). Magnetic biometal-organic framework nanocomposites decorated with folic acid conjugated chitosan as a promising biocompatible targeted theranostic system for cancer treatment. Mater. Sci. Eng. C.

[57] Jalaladdiny S.-S., Badoei-Dalfard A., Karami Z., Sargazi G. (2022). Co-delivery of doxorubicin and curcumin to breast cancer cells by a targeted delivery system based on Ni/Ta core-shell metalorganic framework coated with folic acid-activated chitosan nanoparticles. J. Iran. Chem. Soc..

[58] Elbialy N.S., Aboushoushah S.F., Mohamed N. (2022). Bioinspired synthesis of protein/polysaccharide-decorated folate as a nanocarrier of curcumin to potentiate cancer therapy. Int. J. Pharm..

[59] Zadeh E.S., Ghanbari N., Salehi Z., Derakhti S., Amoabediny G., Akbari M., Tokmedash M.A. (2023). Smart pH-responsive magnetic graphene quantum dots nanocarriers for anticancer drug delivery of curcumin. Mater. Chem. Phys..

[60] Wang Y.J., Tang L., Lu X.H., Liu J.T., Wang Y.Y., Geng H.X., Li X.T., An Q. (2023). Efficacy of epi-1 modified epirubicin and curcumin encapsulated liposomes targeting-EpCAM in the inhibition of epithelial ovarian cancer cells. J. Liposome Res..

[61] Fatih S., Soner C. (2023). Fabrication of curcumin-loaded magnetic PEGylated-PLGA nanocarriers tagged with GRGDS peptide for improving anticancer activity. MethodsX.

[62] Hou J.W., Cong Y.Y., Ji J., Liu Y.X., Hong H., Han X.D. (2024). Spatial targeting of fibrosis-promoting macrophages with nanoscale metal-organic frameworks for idiopathic pulmonary fibrosis therapy. Acta Biomater..

[63] Dai Y.M., Jiang Z.L., Li J.Y., Wang M.F., Liu C., Qi W., Su R.X., He Z.M. (2020). Coassembly of curcumin and a cystine bridged peptide to construct tumorresponsive nano-micelles for efficient chemotherapy. J. Mater. Chem. B.

[64] Han Z.Q., Song B., Yang J.Y., Wang B., Ma Z.Q., Yu L., Li Y.H., Xu H.J., Qiao M.Q. (2022). Curcumin-encapsulated fusion protein-based nanocarrier demonstrated highly efficient epidermal growth factor receptor-targeted treatment of colorectal cancer. J. Agric. Food Chem..

[65] Huang M., Zhai B.T., Fan Y., Sun J., Shi Y.J., Zhang X.F., Zou J.B., Wang J.W., Guo D.Y. (2023). Targeted drug delivery systems for curcumin in breast cancer therapy. Int. J. Nanomed..

[66] Mukerjee A., Ranjan A.P., Vishwanatha J.K. (2016). Targeted nanocurcumin therapy using annexin a2 antibody improves tumor accumulation and therapeutic efficacy against highly metastatic breast cancer. J. Biomed. Nanotechnol..

[67] Jamali Z., Khoobi M., Hejazi S.M., Eivazi N., Abdolahpour S., Imanparast F., Moradi-Sardareh H., Paknejad M. (2018). Evaluation of targeted curcumin (CUR) loaded PLGA nanoparticles for in vitro photodynamic therapy on human glioblastoma cell line. Photodiagnosis Photodyn. Ther..

[68] Demir B., Moulahoum H., Ghorbanizamani F., Barlas F.B., Yesiltepe O., Gumus Z.P., Meral K., Demirkol D.O., Timur S. (2021). Carbon dots and curcumin-loaded CD44-Targeted liposomes for imaging and tracking cancer chemotherapy: A multi-purpose tool for theranostics. J. Drug Deliv. Sci. Technol..

[69] Varshosaz J., Jandaghian S., Mirian M., Sajjadi S.E. (2020). Co-delivery of rituximab targeted curcumin and imatinib nanostructured lipid carriers in non-hodgkin lymphoma cells. J. Liposome Res..

[70] Wang T., Lin M., Mao J., Tian L., Gan H., Hu X., Yan L., Long H., Cai J., Zheng X. (2022). Inflammation-regulated nanodrug sensitizes hepatocellular carcinoma to checkpoint blockade therapy by reprogramming the tumor micro-environment. ACS Appl. Mater. Interfaces.

[71] Nguyen V., You D.G., Kim C.H., Kwon S., Um W., Oh B.H., An J.Y., Jeon J., Park J.H. (2021). An anti-DR5 antibodycurcumin conjugate for the enhanced clearance of activated hepatic stellate cells. Int. J. Biol. Macromol..

[72] Bayer I.S. (2020). Hyaluronic acid and controlled release: A review. Molecules.

[73] Ghalehkhondabi V., Fazlali A., Soleymani M. (2023). Preparation of hyaluronic acid-decorated hollow meso-organosilica/poly (methacrylic acid) nanospheres with redox/pH dual responsivity for delivery of curcumin to breast cancer cells. Mater. Today Chem..

[74] Wang H., Zhang Y.W., Liu Y.R., Ren Y., Wang J.H., Niu B.L., Li W.F. (2022). Preparation of curcumin loaded hyaluronic acid-poly (lactic-co-glycolic acid) micelles with pH response and tumor targeting. Eur. Polym. J..

[75] Zhang Y.T., Xia Q., Li Y.Y., He Z.H., Li Z., Guo T., Wu Z.H., Feng N.P. (2019). CD44 assists the topical anti-psoriatic efficacy of curcumin-loaded hyaluronan-modified ethosomes: A new strategy for clustering drug in inflammatory skin. Theranostics.

[76] Xi Y., Jiang T., Yu Y., Yu J., Xue M., Xu N., Wen J., Wang W., He H., Shen Y. (2019). Dual targeting curcumin loaded alendronate-hyaluronan-octadecanoic acid micelles for improving osteosarcoma therapy. Int. J. Nanomed..

[77] Mokhtari S., Solati-Hashjin M., Khosrowpour Z., Gholipourmalekabadi M. (2021). Layered double hydroxide-galactose as an excellent nanocarrier for targeted delivery of curcumin to hepatocellular carcinoma cells. Appl. Clay Sci..

[78] Gupta B., Sadaria D., Warrier V.U., Kirtonia A., Kant R., Awasthi A., Baligar P., Pal J.K., Yuba E., Sethi G. (2022). Plant lectins and their usage in preparing targeted nanovaccines for cancer immunotherapy. Semin. Cancer Biol..

[79] Hussain N., Jani P.U., Florence A.T. (1997). Enhanced oral uptake of tomato lectin-conjugated nanoparticles in the rat. Pharm. Res..

[80] Sun S.Y., Du X.Y., Fu M.F., Khan A.R., Ji J.B., Liu W.D. (2021). Galactosamine-modified PEG-PLA/TPGS micelles for the oral delivery of curcumin. Int. J. Pharm..

[81] Ghanbari N., Salehi Z., Khodadadi A.A., Shokrgozar M.A., Saboury A.A., Farzaneh F. (2021). Tryptophan-functionalized graphene quantum dots with enhanced curcumin loading capacity and pH-sensitive release. J. Drug Deliv. Sci. Technol..

[82] Guo C.J., Hou X.Y., Liu Y.H., Zhang Y.C., Xu H.Y., Zhao F., Chen D.Q. (2020). Novel Chinese angelica polysaccharide biomimetic nanomedicine to curcumin delivery for hepatocellular carcinoma treatment and immunomodulatory effect. Phytomedicine.

[83] Wang K.L., Guo C.J., Zou S.H., Yu Y.M., Fan X.X., Wang B.B., Liu M.N., Fang L., Chen D.Q. (2018). Synthesis, characterization and in vitro/in vivo evaluation of novel reduction-sensitive hybrid nano-echinus-like nanomedicine. Artif. Cells Nanomed. Biotechnol..

[84] Tian C.H., Asghar S., Hu Z.Y., Qiu Y., Zhang J.W., Shao F., Xiao Y.Y. (2019). Understanding the cellular uptake and biodistribution of a dual-targeting carrier based on redox-sensitive hyaluronic acid-ss-curcumin micelles for treating brain glioma. Int. J. Biol. Macromol..

[85] Dong X., Zou S.H., Guo C.J., Wang K.L., Zhao F., Fan H.Y., Yin J.G., Chen D.Q. (2017). Multifunctional redoxresponsive and cd44 receptor targeting polymer-drug nanomedicine based curcumin and alendronate: Synthesis, characterization and in vitro evaluation. Artif. Cells Nanomed. Biotechnol..

[86] Malekmohammadi S., Hadadzadeh H., Rezakhani S., Amirghofran Z. (2019). Design and synthesis of gatekeeper coated dendritic silica/titania mesoporous nanoparticles with sustained and controlled drug release properties for targeted synergetic chemo-sonodynamic therapy. ACS Biomater. Sci. Eng..

[87] Telang N. (2022). Drug-resistant stem cells: Novel approach for colon cancer therapy. Int. J. Mol. Sci..

[88] Zhang Y.F., Wang Y.R., Lu Y., Quan H., Wang Y.Q., Song S.J., Guo H.Y. (2025). Advanced oral drug delivery systems for gastrointestinal targeted delivery: The design principles and foundations. J. Nanobiotechnol..

[89] Shao H., Liu M., Jiang H., Zhang Y. (2025). Polysaccharide-based drug delivery targeted approach for colon cancer treatment: A comprehensive review. Int. J. Biol. Macromol..

[90] Chen M., Lan H.R., Jin K.T., Chen Y. (2023). Responsive nanosystems for targeted therapy of ulcerative colitis: Current practices and future perspectives. Drug Deliv..

[91] Tian Y., Hu Q., Sun Z., Yu Y., Li X., Tian T., Bi X., Li Y., Niu B., Zhang Z. (2024). Colon targeting pH-responsive coacervate microdroplets for treatment of ulcerative colitis. Small.

[92] Khatik R., Mishra R., Verma A., Dwivedi P., Kumar V., Gupta V., Paliwal S.K., Mishra P.R., Dwivedi A.K. (2013). Colon specific delivery of curcumin by exploiting Eudragit-decorated chitosan nanoparticles in vitro and in vivo. J. Nanoparticle Res..

[93] Lertpairod J., Tiyaboonchai W. (2022). pH-sensitive beads containing curcumin loaded nanostructured lipid carriers for a colon targeted oral delivery system. J. Pharm. Investig..

[94] Beloqui A., Coco R., Memvanga P.B., Ucakar B., Rieux A.D., Préat V. (2014). pH sensitive nanoparticles for colonic delivery of curcumin in inflammatory bowel disease. Int. J. Pharmacol..

[95] Mashaqbeh H., Obaidat R., Alsmadi M.M., Bardaweel S., Hailat N. (2024). Characterization and optimization of colon specific nanosponges immobilized polymeric microbeads formulation for the combined delivery of 5-fluorouracil and curcumin. J. Drug Deliv. Sci. Technol..

[96] Li H., Gao Z.X., Xu J.J., Sun W., Wu J.R., Zhu L., Gao M.J., Zhan X.B. (2022). Encapsulation of polyphenols in pH-responsive micelles self-assembled from octenyl-succinylated curdlan oligosaccharide and its effect on the gut microbiota. Colloids Surf. B Biointerfaces.

[97] Moideen M.M.J., Karuppaiyan K., Kandhasamy R., Seetharaman S. (2019). Skimmed milk powder and pectin decorated solid lipid nanoparticle containing soluble curcumin used for the treatment of colorectal cancer. J. Food Process Eng..

[98] Zhang G.S., Han W., Zhao P.X., Wang Z.J., Li M., Sui X.F., Liu Y.H., Tian B.C., He Z.G., Fu Q. (2023). Programmed pHresponsive core-shell nanoparticles for precisely targeted therapy of ulcerative colitis. Nanoscale.

[99] Guo X., Liu H.Y., Hou R.Y., Chen G.J., Xiao H., Liu L.Y., Ciftci O.N., Liu L.L. (2024). Design strategies of polysaccharide, protein and lipid-based nano-delivery systems in improving the bioavailability of polyphenols and regulating gut homeostasis. Int. J. Biol. Macromol..

[100] Huang D., Zou M.L., Xu C.L., Wang Y.M., Xu Z.J., Zhang W.C., Tang S.J., Weng Z.Q. (2024). Colon-targeted oral delivery of hydroxyethyl starch–curcumin microcapsules loaded with multiple drugs alleviates DSS-induced ulcerative colitis in mice. Macromol. Biosci..

[101] Li S.Y., Jin M.F., Wu Y.H., Jung S., Li D.D., He N.N., Lee M.S. (2021). An efficient enzyme-triggered controlled release system for colon-targeted oral delivery to combat dextran sodium sulfate (DSS)-induced colitis in mice. Drug Deliv..

[102] Zhang C., Chen Z.J., He Y.N., Xian J., Luo R.F., Zheng C., Zhang J.M. (2021). Oral colon-targeting core-shell microparticles loading curcumin for enhanced ulcerative colitis alleviating efficacy. Chin. Med..

[103] Zeybek N., Buyukkileci A.O., Gulec S., Polat M., Polat H. (2023). Designing robust xylan/chitosan composite shells around drug-loaded MSNs: Stability in upper GIT and degradation in the colon microbiota. J. Drug Deliv. Sci. Technol..

[104] Wen Z., Kang L., Fu H., Zhu S., Ye X., Yang X., Zhang S., Hu J., Li X., Chen L. (2023). Orall delivery of porous starch-loaded bilayer microgels for controlled drug delivery and treatment of ulcerative colitis. Carbohydr. Polym..

[105] Meng F.B., Zhang Q., Li Y.C., Li J.J., Peng L.X. (2020). Konjac glucomannan octenyl succinate as a novel encapsulation wall material to improve curcumin stability and bioavailability. Carbohydr. Polym..

[106] Wang L.H., Xiao J.X., Li X.D., Huang G.Q. (2021). Carboxymethyl konjac glucomannan coating on multilayered emulsions for improved bioavailability and targeted delivery of curcumin. Food Funct..

[107] Wang Z.D., Peng H.H., Guan Y.X., Yao S.J. (2022). Supercritical CO_2_ assisted micronization of curcumin-loaded oil-inwater emulsion promising in colon targeted delivery. J. CO2 Util..

[108] Kotla N.G., Burke O., Pandit A., Rochev Y. (2019). An Orally administrated hyaluronan functionalized polymeric hybrid nanoparticle system for colon-specific drug delivery. Nanomaterials.

[109] Borah P.K., Das A.S., Mukhopadhyay R., Sarkar A., Duary R.K. (2020). Macromolecular design of folic acid functionalized amylopectin-albumin core-shell nanogels for improved physiological stability and colon cancer cell targeted delivery of curcumin. J. Colloid Interface Sci..

[110] Li H., He W.J., Wang Z.J., Zhang Q., Hu D., Ding K., Xie Q.T., Xu Y.Q., Shan Y., Ding S.H. (2025). Improving the prebiotic activity and oxidative stability of carboxymethyl curdlan-quercetin conjugates stabilized Pickering emulsions for the colonic targeting delivery of curcumin. Food Res. Int..

[111] Izadi Z., Rashidi M., Derakhshankhah H., Dolati M., Kermanshahi M.G., Adibi H., Samadian H. (2023). Curcumin-loaded porous particles functionalized with pH-responsive cell-penetrating peptide for colorectal cancer targeted drug delivery. RSC Adv..

[112] Yang J., Chen X.X., Lin J.Q., Shen M.Y., Wang Y.X., Sarkar A., Wen Hl Xie J.H. (2024). Co-delivery of resveratrol and curcumin based on Mesona chinensis polysaccharides/zein nanoparticle for targeted alleviation of ulcerative colitis. Food Biosci..

[113] Zeybek N., Polat H., Gulec S., Buyukkileci A.O. (2025). Development of xylan-coated acid-resistant micellar drug carriers for colon-targeted oral delivery. Int. J. Polym. Mater..

[114] Zhang C., Li J.X., Xiao M., Wang D., Qu Y., Zou L., Zheng C.A., Zhang J.M. (2022). Oral colon-targeted mucoadhesive micelles with enzyme-responsive controlled release of curcumin for ulcerative colitis therapy. Chin. Chem. Lett..

[115] Hua Y.J., Wei Z.H., Xue C.H., Si J.Y. (2024). Stability and programmed sequential release of Lactobacillus plantarum and curcumin encapsulated in bilayer-stabilized W1/O/W2 double emulsion: Effect of pectin as protective shell. Int. J. Biol. Macromol..

[116] Wu M., Ping H., Wang K., Ding H., Zhang M., Yang Z., Du Q. (2023). Oral delivery of pectin-chitosan hydrogels entrapping macrophage-targeted curcumin-loaded liposomes for the treatment of ulcerative colitis. Int. J. Pharm..

[117] Huang D., Wang Y., Xu C., Zou M., Ming Y., Luo F., Xu Z., Miao Y., Wang N., Lin Z. (2024). Colon-targeted hydroxyethyl starch-curcumin microspheres with high loading capacity ameliorate ulcerative colitis via alleviating oxidative stress, regulating inflammation, and modulating gut microbiota. Int. J. Biol. Macromol..

[118] Kurra P., Narra K., Orfali R., Puttugunta S.B., Alam Khan S., Meenakshi D.U., Francis A.P., Asdaq S.M.B., Imran M. (2022). Studies on jackfruit-okra mucilage-based curcumin mucoadhesive tablet for colon targeted delivery. Front. Pharmacol..

[119] Hales D., Muntean D.-M., Neag M.A., Kiss B., Ștefan M.-G., Tefas L.R., Tomuță I., Sesărman A., Rațiu I.-A., Porfire A. (2022). Curcumin-loaded microspheres are Effective in preventing oxidative stress and intestinal inflammatory abnormalities in experimental ulcerative colitis in rats. Molecules.

[120] Cai R., Pan S.Y., Li R.X., Xu X.Y., Pan S.Y., Liu F.X. (2022). Curcumin loading and colon release of pectin gel beads: Effect of different de-esterification method. Food Chem..

[121] Liang D.S., Shen X.F., Li T.T., Deng Y., Xiao H.L., Li Z.P., Bai S.C., Ma X.Y., Liao X.P., Zhao D.H. (2025). A bionic yeast for the colon-targeted delivery of curcumin in the treatment of inflammatory bowel disease. Chem. Eng. J..

[122] Sun X., Wang N., Yang L.Y., Ouyang X.K., Huang F. (2019). Folic acid and PEI modified mesoporous silica for targeted delivery of curcumin. Pharmaceutics.

[123] Wang Z., Deng X.P., Ding J.S., Zhou W.H., Zheng X., Tang G.T. (2018). Mechanisms of drug release in pH-sensitive micelles for tumor targeted drug delivery system: A review. Int. J. Pharm..

[124] Emami J. (2006). In vitro-in vivo correlation: From theory to applications. J. Pharm. Pharm. Sci..

[125] Feng K., Li C., Wei Y.S., Zong M.H., Han S.Y. (2019). Development of a polysaccharide based multi-unit nanofiber mat for colon-targeted sustained release of salmon calcitonin. J. Colloid Interface Sci..

[126] Bokkhim H., Bansal N., Grondahl L., Bhandari B. (2015). In-vitro digestion of different forms of bovine lactoferrin encapsulated in alginate micro-gel particles. Food Hydrocoll..

[127] Li C., Wei Y.S., Wen P., Feng K., Zong Z.M., Wu H. (2018). Preparation and characterization of an electrospun colonspecific delivery system for salmon calcitonin. RSC Adv..

[128] Singh S.K., Yadav A.K., Prudhviraj G., Gulati M., Kaur P., Vaidya Y. (2015). A novel dissolution method for evaluation of polysaccharide based colon specific delivery systems: A suitable alternative to animal sacrifice. Eur. J. Pharm. Sci..

[129] Mousazadeh H., Yazdani Y., Mohammadi Z., Alivirdiloo V., Nikzad B., Mohammadzadeh M. (2024). Fabrication of poly (lactic-co-glycolic acid)/mesoporous silica composite nanofibers for controllable co-delivery of 5-fluorouracil and curcumin against HT-29 colon cancer cells. J. Mater. Sci..

[130] Abouaitah K., Swiderska-Sroda A., Farghali A.A., Wojnarowicz J., Lojkowski W. (2018). Folic acid-conjugated mesoporous silica particles as nanocarriers of natural prodrugs for cancer targeting and antioxidant action. Oncotarget.

[131] Duan D.Y., Wang A.P., Ni L., Zhang L.P., Yan X.J., Jiang Y., Mu H.J., Wu Z.M., Sun K.X., Li Y.X. (2018). Trastuzumaband Fab’ fragment-modified curcumin PEG-PLGA nanoparticles: Preparation and evaluation in vitro and in vivo. Int. J. Nanomed..

[132] Feng R.L., Deng P.Z., Song Z.M., Chu W., Zhu W.X., Teng F.F., Zhou F.L. (2017). Glycyrrhetinic acid-modified PEG-PCL copolymeric micelles for the delivery of curcumin. React. Funct. Polym..

[133] Chen G.Q., Li J.L., Cai Y.B., Zhan J., Gao J., Song M.C., Shi Y., Yang Z.M. (2017). A glycyrrhetinic acid-modified curcumin supramolecular hydrogel for liver tumor targeting therapy. Sci. Rep..

[134] Nandy B.C., Verma V., Dey S., Mazumder B. (2014). Three levels face centered central composite design of colon targeted micro-particulates system of celecoxib: Screening of vehicles variables and in vivo studies. Curr. Drug Del..

[135] Sawarkar S.P., Deshpande S.G., Bajaj A.N., Soni P.S., Nikam V.S. (2019). Potential of low molecular weight natural polysaccharides for colon targeted formulation and its evaluation in human by gamma scintigraphy. J. Pharm. Investig..

[136] Handali S., Moghimipour E., Kouchak M., Ramezani Z., Dorkoosh F.A. (2018). In vitro and in vivo evaluation of coated capsules for colonic delivery. J. Drug Deliv. Sci. Technol..

[137] Du B., Du Q., Bai Y.M., Yu L.L., Wang Y.H., Huang J., Zheng M., Shen G.P., Zhou J., Yao H.C. (2020). Chemotherapy based on “domino-effect” combined with immunotherapy amplifying the efficacy of an anti-metastatic treatment. Mater. Chem. B.

[138] Yallapu M.M., Khan S., Maher D.M., Ebeling M.C., Sundram V., Chauhan N., Ganju A., Balakrishna S., Gupta B.K., Zafar N. (2014). Anti-cancer activity of curcumin loaded nanoparticles in prostate cancer. Biomaterials.

[139] Liu J., Zhang Y., Zhou J., Yang C., Wang W., Chu L., Huang F., Deng L., Kong D., Liu J. (2016). Folic acid-targeted disulfide-based cross-linking micelle for enhanced drug encapsulation stability and site-specific drug delivery against tumors. Int. J. Nanomed..

[140] Langhans S.A. (2018). Three-dimensional in vitro cell culture models in drug discovery and drug repositioning. Front. Pharmacol..

[141] Nguyen T.L.A., Vieira-Silva S., Liston A., Raes J. (2015). How informative is the mouse for human gut microbiota research?. Dis. Model. Mech..

[142] Drost J., Clevers H. (2018). Organoids in cancer research. Nat. Rev. Cancer.

[143] Shah P., Fritz J.V., Glaab E., Desai M.S., Greenhalgh K., Frachet A., Niegowska M., Estes M., Jäger C., Seguin-Devaux C. (2016). A microfluidics-based in vitro model of the gastrointestinal human-microbe interface. Nat. Commun..

[144] Huang X., Yu Y., Tian N., Huang J., Zhang X., Yu R. (2025). Human microbiota-associated animal models: A review. Front. Cell. Infect. Microbiol..

[145] Rago V., Perri A., Di Agostino S. (2023). New Therapeutic perspectives in prostate cancer: Patient-derived organoids and patient-derived xenograft models in precision medicine. Biomedicines.

[146] Ranjit R. (2025). Advancing Monoclonal Antibody Manufacturing: Process Optimization, Cost Reduction Strategies, and Emerging Technologies. Biol. Targets Ther..

[147] Wagh A., Song H.T., Zeng M., Tao L., Das T.K. (2018). Challenges and new frontiers in analytical characterization of antibody-drug conjugates. mAbs.

[148] Ben Mkaddem S., Benhamou M., Monteiro R.C. (2019). Understanding Fc receptor involvement in inflammatory diseases: From mechanisms to new therapeutic tools. Front. Immunol..

[149] Sripetthong S., Nalinbenjapun S., Basit A., Surassmo S., Sajomsang W., Ovatlarnporn C. (2023). Preparation of self-assembled, curcumin-loaded nano-micelles using quarternized chitosan-vanillin imine (QCS-Vani Imine) conjugate and evaluation of synergistic anticancer effect with cisplatin. Funct. Biomater..

[150] Ndeh D.A., Nakjang S., Kwiatkowski K.J., Sawyers C., Koropatkin N.M., Hirt R.P., Bolam D.N. (2025). A Bacteroides thetaiotaomicron genetic locus encodes activities consistent with mucin O-glycoprotein processing and N-acetylgalactosamine metabolism. Nat. Commun..

[151] Zhou R., Yang H.J., Zhu P., Liu Y.J., Zhang Y.J., Zhang W., Zhou H.H., Li X., Li Q. (2023). Effect of gut microbiota on the pharmacokinetics of nifedipine in spontaneously hypertensive rats. Pharmaceutics.

[152] Ranjan A.P., Mukerjee A., Vishwanatha J.K. (2010). Solid in Oil/Water Emulsion-Diffusion-Evaporation Formulation for Preparing Curcumin-Loaded PLGA Nanoparticles. U.S. Patent.

[153] Sinko P.J., Gao J., Deshmukh M., Zhang X., Palombo M.S., Ibrahim S. (2012). Synergistic Combinations to Reduce Particle Dose for Targeted Treatment of Cancer and Its Metastases. U.S. Patent.

[154] Chauhan S., Jaggi M., Yallapu M.M. (2013). Magnetic Nanoparticle Formulations, Methods for Making Such Formulations, and Methods for Their Use. U.S. Patent.

[155] Braden A.R.C., Vishwanatha J.K. (2015). Formulation of Active Agent Loaded Activated PLGA Nanoparticles for Targeted Cancer Nano-Therapeutics. U.S. Patent.

[156] Chang R., Sun L., Webster T.J., Mi G. (2017). Amphiphilic Peptide Nanoparticles for Use as Hydrophobic Drug Carriers and Antibacterial Agents. U.S. Patent.

[157] Huang W.T., Chiang Y.C., Liu D.M. (2018). Nanocomposite, a Preparation Method Thereof and Method for Treating Cancer Using the Same. U.S. Patent.

[158] Yin J., Qiao Z., Liu H., Mao X., Zha J. (2019). Preparation and Application of a Reactive Oxygen Species-Responsive Polycurcumin Prodrug-Based Nanocarrier with Ultrahigh Drug Loading Capacity.

[159] Wen R., Feng L., Lan Y., Liu Y. (2020). A Nanocarrier Material for Co-Delivery of Curcumin and miRNA in Prostate Cancer Therapy with GSH Responsiveness and CT Imaging Capability.

[160] Zhang H.-G. (2021). Edible Plant Exosome-like Nanovectors for Vaccination. U.S. Patent.

[161] Li G.-C., Huang X.-Z., Lü L., Shi Y.-H., Xu J.-J., Mao Y.-P., Meng N., Deng Z.-C. (2023). Amphiphilic Block Copolymer-Based Nano-Carrier Simultaneously Targeting Tumor Cells and Cancer Stem Cells.

[162] Liu Y., Chen Z., Zhang X., Li H., Yan Z., Wu T. (2023). Targeted Nano-Carrier System Constructed from Dopamine Derivatives for Enhanced Delivery of Hydrophobic Drugs. Chinese Patent.

[163] Sun C.C., Liu D.M. (2024). Injectable Nanocomposite Gel Composition for Co-Delivery of Multiple Medicines or Drugs. U.S. Patent.

[164] Xiao J.X., Huang G.Q., Cao Y.Q., Li K.Y. (2021). Curcumin-Loaded Pickering Emulsion with Colon-Targeted Delivery Function and Preparation Method and Application Thereof. Chinese Patent.

[165] Xiao J.X., Wang L.H., Huang G.Q., Zhang X.R. (2021). Curcumin Double-Layer Emulsion with Colon-Targeted Delivery Function and Preparation Method and Application Thereof. Chinese Patent.

[166] Zhan X.B., Li H., Zhu L., Gao M.J., Jiang Y. (2024). pH- and Microbe-Dual-Responsive Colon-Targeted Micelles and Preparation Method Thereof. Chinese Patent.

[167] Li B., Yuan Y., Zhang Y., Li L., Zhang X., Guo Q.Y., Zheng Q.S. (2023). Colon-Targeted Oil-in-Water Pickering Emulsion Based on Shellac Nanoparticles and Chitosan and Its Preparation and Application. Chinese Patent.

